# Doping-Engineered Proangiogenic Nanozymes Orchestrate Ischemic Tissue Regeneration via Cytoprotection and Revascularization

**DOI:** 10.34133/research.1260

**Published:** 2026-04-28

**Authors:** Huili Li, Xiaosheng Sheng, Chuandong Qiu, Dongmin Chen, Lefeng Su, Qishu Jin, Xin Gong, Yanxin Zhang, Zhaoxu Meng, Xin Li, Hangbin Xia, Chunlong Zhang, Zhen Zeng, Xiaoting Chen, Jiehao Chen, Yiren Jiao, Jiang Chang, Yumei Que, Zhaowenbin Zhang, Chen Yang, Wenzhong Li

**Affiliations:** ^1^ School of Ophthalmology and Optometry, School of Biomedical Engineering, Wenzhou Medical University, Wenzhou, Zhejiang 325027, China.; ^2^ Joint Centre of Translational Medicine, The First Affiliated Hospital of Wenzhou Medical University, Wenzhou, Zhejiang 325000, China.; ^3^ Zhejiang Engineering Research Center for Tissue Repair Materials, Wenzhou Institute, University of Chinese Academy of Sciences, Wenzhou, Zhejiang 325000, China.; ^4^ Department of Cardiology, Jinhua People’s Hospital, Jinhua, Zhejiang 321000, China.; ^5^ Orthopedic Institute, Suzhou Medical College, Soochow University, Suzhou, Jiangsu 215000, China.; ^6^ Department of Cell Biology, Third Military Medical University, Chongqing, China.; ^7^ The Affiliated Hospital of Southwest Jiaotong University, The Third People’s Hospital of Chengdu, Chengdu, Sichuan 610031, China.; ^8^ Animal Experimental Center, West China Hospital, Sichuan University, Chengdu, Sichuan 610213, China.; ^9^ State Key Laboratory of Advanced Fiber Materials, College of Biological Science and Medical Engineering, Donghua University, Shanghai 201620, China.; ^10^ National Engineering Research Center of Ophthalmology and Optometry, Eye Hospital of Wenzhou Medical University, Wenzhou, Zhejiang 325027, China.; ^11^ National Clinical Research Center for Ocular Diseases, Eye Hospital, Wenzhou Medical University, Wenzhou, Zhejiang 325027, China.

## Abstract

Effective therapy for ischemic diseases requires not only timely restoration of perfusion but also protection of ischemic tissues from secondary insults, particularly oxidative-stress-induced injury. Here, we propose the concept of “proangiogenic nanozymes” and develop an efficient screening platform by doping angiogenic elements (Mg, Co, Cu, Zn, Sr, or Eu) into an archetypal antioxidative nanozyme, Prussian blue. Systematic assessment of catalytic activity and proangiogenic performance identified Cu-doped Prussian blue (CuPB) as the lead candidate. In addition to efficiently decomposing multiple reactive oxygen species, thereby attenuating oxidative stress, reducing apoptosis, and protecting ischemic tissues from secondary injury, CuPB nanozymes also stimulated angiogenesis, thereby accelerating tissue repair. In murine models of hind limb ischemia and myocardial infarction, CuPB conferred therapeutic benefits after both local and systemic administration, underscoring its translational potential. This proangiogenic nanozyme strategy offers an integrated and effective approach to ischemic tissue regeneration, bridging catalytic nanomedicine and vascular repair.

## Introduction

Ischemic diseases are prevalent, life-threatening disorders that affect diverse organ systems. Representative examples include peripheral artery disease (PAD) leading to acute limb ischemia, ischemic heart disease (IHD) manifesting as myocardial infarction (MI), and ischemic cerebrovascular disease presenting as ischemic stroke [1–3]. These disorders frequently inflict irreversible injury on vital organs, such as the heart and brain, and in severe cases are fatal, ranking among the foremost causes of global mortality [4]. Although clinical interventions such as vascular interventional therapy, coronary artery bypass grafting, and thrombolysis have markedly improved survival, their primary objective is rapid restoration of blood supply, which is essential for salvaging ischemic tissue [3,5,6]. However, reperfusion alone is far from sufficient. Beyond the risk of ischemia–reperfusion injury, which exacerbates oxidative stress and inflammation, thereby causing secondary iatrogenic damage, the irreversible injury incurred during the ischemic phase, together with the challenges of repair and regeneration [7,8], remains the principal determinant of long-term prognosis and represents an unmet clinical need.

Despite differences in etiology and severity across organ systems, ischemic diseases share convergent pathophysiological mechanisms [9,10]. Fundamentally, inadequate blood supply leads to tissue hypoxia, mitochondrial dysfunction, and cell death (with apoptosis as the typical form), which in turn drive excessive production of reactive oxygen species (ROS). This amplifies oxidative stress and triggers inflammatory cascades that accelerate secondary necrosis [9,10]. Reperfusion further aggravates injury through additional ROS bursts and inflammatory amplification, worsening tissue degeneration and functional loss [7,8]. Conventional therapeutic approaches, such as antioxidants to scavenge ROS or anti-inflammatory agents to suppress immune overactivation, aim to protect viable cells but typically yield only transient benefits [11]. Beyond mitigating oxidative stress, inflammation, and apoptosis, timely induction of angiogenesis at the injury site, which restores perfusion and nutrient delivery, is equally critical for functional recovery [12]. Accordingly, advanced biological strategies such as growth factor delivery, gene therapy, and cell-based interventions have been explored [13]. Yet, their clinical translation remains hindered by complex manufacturing, poor in vivo stability, and high costs [14,15]. Thus, a therapeutic platform capable of simultaneously scavenging ROS and actively promoting angiogenesis to both protect ischemic tissue and restore vascular perfusion would be highly desirable.

Nanozymes, an emerging class of catalytic nanomaterials with intrinsic enzyme-like activities, have shown promise in addressing these challenges. With inherent ROS-scavenging capabilities, high stability, and tunable composition, nanozymes can continuously neutralize oxidative stress and modulate inflammation in ischemic tissues [16]. Among them, Prussian blue (PB) nanozymes are particularly attractive owing to their excellent biocompatibility and multienzyme-mimetic properties, including superoxide dismutase (SOD)- and catalase (CAT)-like activities [17,18]. These features enable efficient ROS detoxification and attenuation of oxidative injury, with multiple studies reporting significant reductions in ischemic damage and inflammation in cardiovascular models [19,20]. However, most nanozymes developed to date have been restricted to antioxidative and immunomodulatory functions, without the capacity to directly stimulate angiogenesis. The absence of active revascularization severely limits long-term functional recovery, as the mere reduction of cell death, without concomitant revascularization, is insufficient to restore perfusion. Equipping nanozymes with proangiogenic functionality alongside ROS scavenging could therefore represent a paradigm shift in ischemic tissue therapy. Yet, to date, no nanozyme platform has been explicitly designed to integrate these dual functions.

Ion doping engineering offers a rational strategy to modulate the biological activities of nanozymes. Several bioactive ions have been demonstrated to promote angiogenesis via distinct pathways. To systematically identify the optimal dopant for regulating vascular regeneration, we selected a library of 6 representative metal ions (Mg, Co, Cu, Zn, Sr, and Eu) based on their distinct yet complementary angiogenic mechanisms and their chemical compatibility with the PB crystal lattice. Specifically, these ions target diverse signaling pathways essential for angiogenesis: Upstream regulation: Co and Eu ions stabilize hypoxia-inducible factor-1α (HIF-1α), acting as “hypoxia mimetics” to up-regulate vascular endothelial growth factor (VEGF) and fibroblast growth factor (FGF) [21,22]. Signaling activation: Mg and Sr ions activate the phosphoinositide 3-kinase/protein kinase B (PI3K/Akt) and Wnt/β-catenin pathways, stimulating NO and VEGF production [23–26]. Enzyme modulation: Zn ions stabilize the dimeric structure of endothelial nitric oxide synthase (eNOS), promoting lumen formation [27]. Multistage stimulation: Cu ions act broadly across multiple stages of vascular growth, from endothelial proliferation to matrix remodeling [28,29]. Furthermore, the porous metal–organic framework of PB allows for the flexible substitution of iron nodes with these transition metal ions without collapsing the crystal structure. Therefore, by screening this specific panel of bioactive ions, we aimed to develop a dual-functional “proangiogenic nanozyme” that effectively couples catalytic ROS detoxification with targeted vascular regenerative cues.

**Fig. 1.**
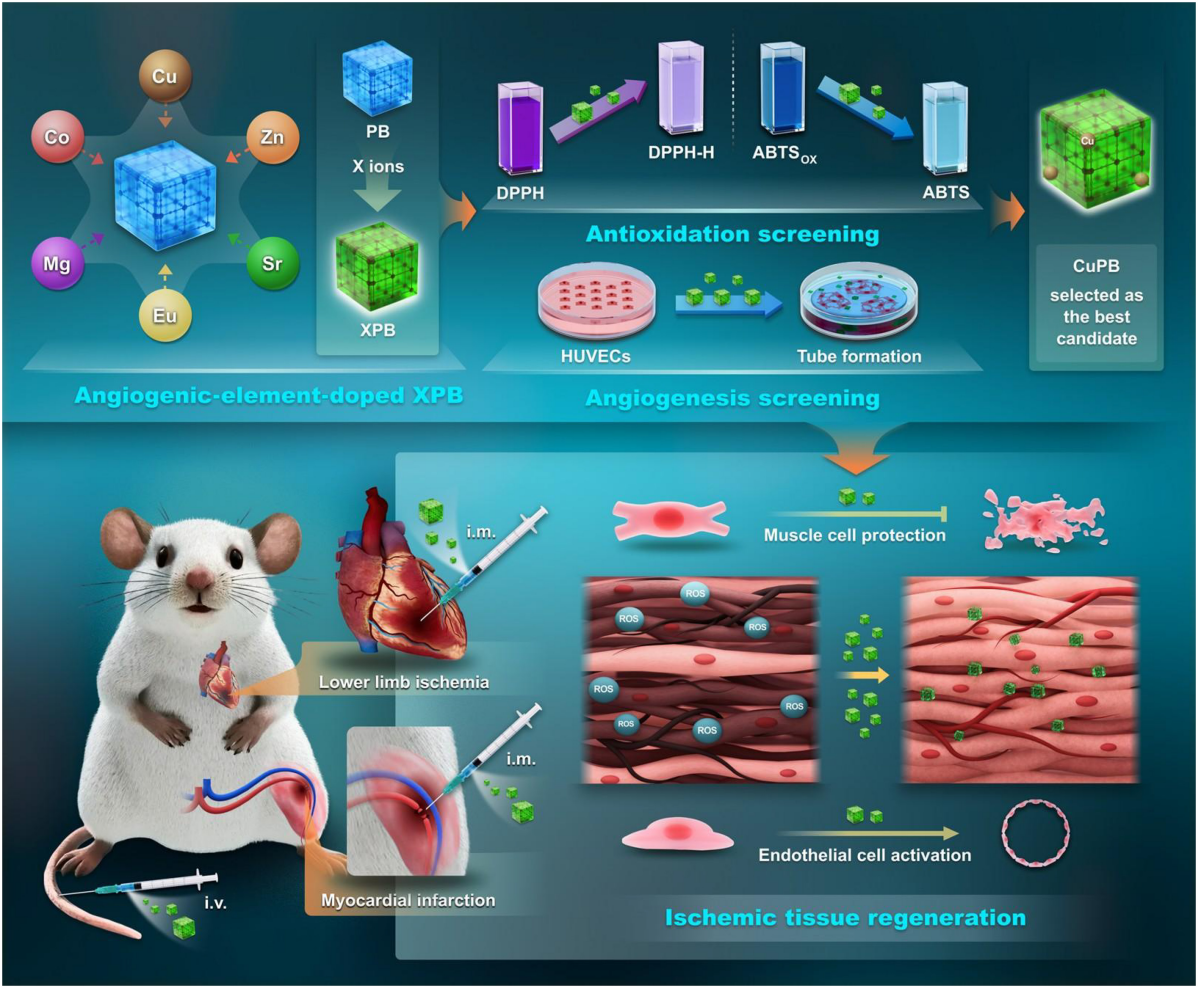
Conception of “proangiogenic nanozymes” for ischemic tissue regeneration. proangiogenic nanozymes were generated by incorporating angiogenic X ions (X = Mg, Co, Cu, Zn, Sr, and Eu) into Prussian blue (PB) nanozymes, followed by systematic screening of the resulting XPB formulations to identify the most effective candidate with superior antioxidative and proangiogenic activities. The selected XPB nanozyme integrates potent cytoprotective effects with robust vascular regeneration, thereby promoting efficient tissue repair in representative models of limb ischemia and myocardial infarction.

Here, we propose and implement this concept by incorporating angiogenic X ions (X = Mg, Co, Cu, Zn, Sr, and Eu) into PB nanozymes, systematically screening the resulting XPB formulations for antioxidative and proangiogenic performance, and Cu-doped PB (CuPB) was identified as the lead candidate (Fig. 1). The selected CuPB nanozyme was comprehensively characterized for structure, composition, and catalytic activity, as well as for cytoprotective effects in C2C12 myoblasts and H9c2 cardiomyocytes and angiogenic stimulation in human umbilical vein endothelial cells (HUVECs). Finally, 2 murine models of ischemic injury, critical limb ischemia (CLI) and MI, were employed to evaluate therapeutic efficacy following both intramuscular and intravenous delivery. This work pioneers the integration of angiogenic ion doping into nanozyme design, bridging catalytic nanomedicine with vascular regenerative therapy. The resulting XPB nanozyme addresses the critical bottleneck of insufficient vascularization in current nanozyme-based strategies and offers a versatile and translationally promising approach for ischemic tissue repair.

## Results

### Synthesis and screening of proangiogenic XPB nanozymes

**Fig. 2.**
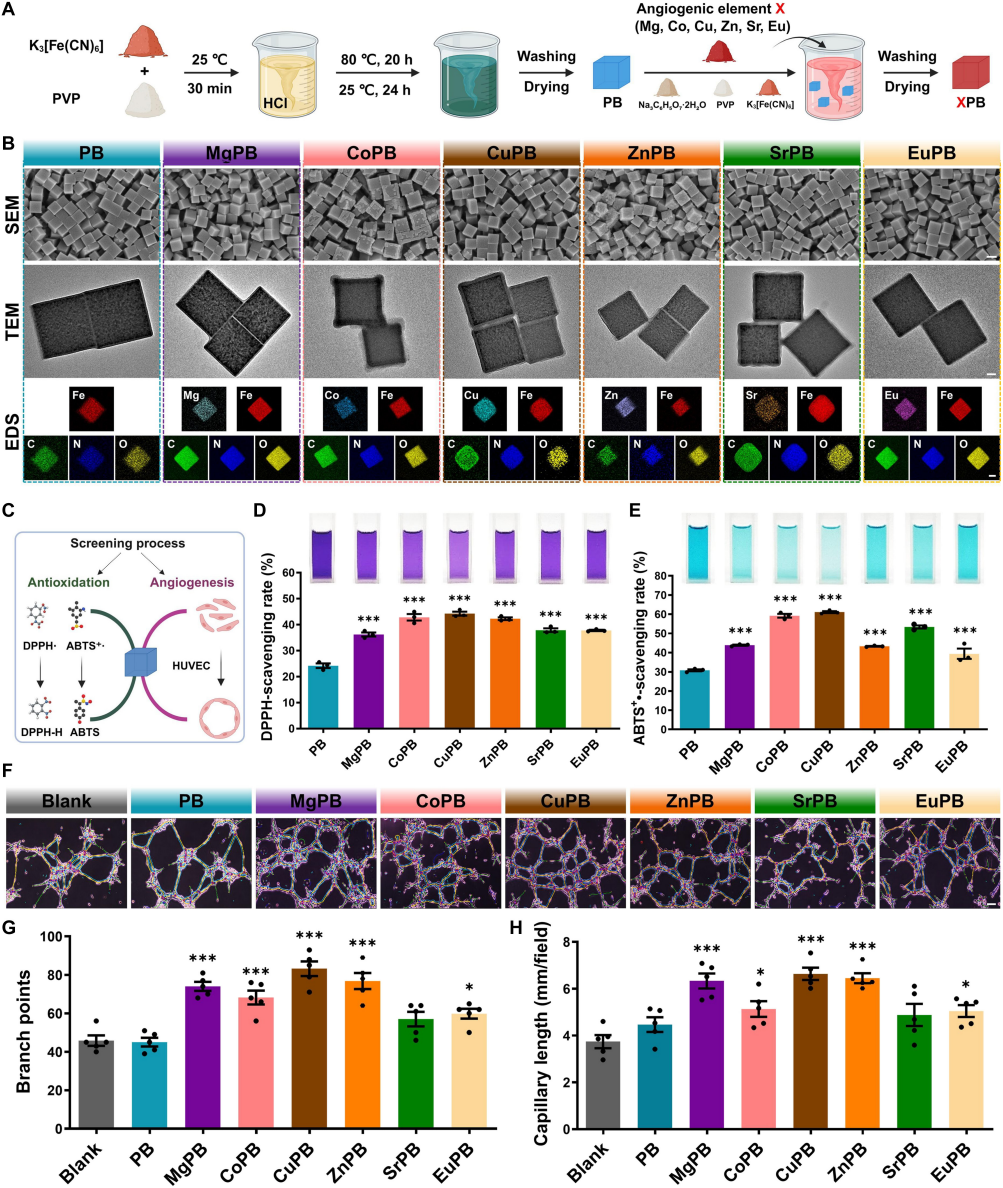
Synthesis and screening of proangiogenic XPB nanozymes. (A) Schematic illustration depicting the synthesis process of XPB nanozymes. (B) Representative microscopic characterizations of XPB nanozymes, including scanning electron microscopy (SEM) images (scale bar: 100 nm), transmission electron microscopy (TEM) images (scale bar: 25 nm), and energy-dispersive x-ray spectroscopy (EDS) elemental mapping (scale bar: 50 nm). (C) Schematic workflow for systematic screening of antioxidative and proangiogenic performance of XPB nanozymes. (D and E) DPPH- and ABTS^+^•-radical-scavenging activities of XPB nanozymes. *n* = 3. DPPH, 1-diphenyl-2-picrylhydrazyl; ABTS, 2,2′-azinobis(3-ethylbenzothiazoline-6-sulfonic acid). (F) In vitro proangiogenic capability of XPB nanozymes assessed by human umbilical vein endothelial cell (HUVEC) tube-formation assays. Scale bar: 100 μm. (G) Quantification of vascular branch points. *n* = 5. (H) Quantification of total vascular length. *n* = 5. (A) and (C) were created with BioRender.com. * indicates significant difference compared to the Prussian blue (PB) group. *P < 0.05 and ***P < 0.001.

PB nanozymes were synthesized by a modified coprecipitation route using potassium ferricyanide (K_4_[Fe(CN)_6_]) as the structural precursor. XPB (X = Mg, Co, Cu, Zn, Sr, or Eu) nanozymes were subsequently obtained by introducing angiogenic X elements through the addition of the corresponding chloride salts during synthesis (Fig. 2A). Scanning electron microscopy and transmission electron microscopy images revealed that both PB and XPB nanoparticles exhibited a uniform cubic morphology. Energy-dispersive x-ray spectroscopy elemental mapping further demonstrated the homogeneous distribution of the doped elements within the framework, confirming the successful preparation of XPB nanozymes (Fig. 2B). Dynamic light scattering analysis confirmed that both PB and XPB nanoparticles were uniformly dispersed in aqueous solution, exhibiting average hydrodynamic diameters in the range of 234.7 ± 8.3 to 288.1 ± 7.7 nm (Fig. S1 and Table S1), consistent with scanning electron microscopy observations. Inductively coupled plasma mass spectrometry (ICP-MS) was employed to quantify the doping content of each element in the XPB nanozymes. The weight ratios of doped elements to Fe were maintained within the range of 7% to 9%, with no statistically significant variation among different formulations (Fig. S2).

To systematically evaluate the antioxidant capacity and angiogenic potential of XPB nanozymes, 2 classic radical-scavenging assays, namely, 1,1-diphenyl-2-picrylhydrazyl (DPPH) and 2,2′-azinobis(3-ethylbenzothiazoline-6-sulfonic acid) (ABTS) were performed for antioxidant screening, together with a HUVEC tube-formation assay for angiogenesis screening, respectively (Fig. 2C). In the DPPH assay, all XPB formulations exhibited significantly enhanced radical-scavenging ability compared with PB, with Co-doped PB (CoPB) and CuPB showing the highest activities, at 1.77- and 1.82-fold greater than that of PB, respectively (Fig. 2D). Similar trends were observed in the ABTS assay, where CoPB and CuPB again demonstrated the greatest catalytic efficiencies, with scavenging ratios 1.92- and 1.98-fold higher than that of PB, respectively (Fig. 2E). We speculate this enhanced catalytic activity to the mixed valence states (Cu^+^/Cu^2+^ and Co^2+^/Co^3+^) introduced during doping, which facilitate electron transfer and thereby boost catalytic kinetics. For in vitro angiogenesis assessment, HUVEC viability was first evaluated to determine a noncytotoxic working concentration. PB nanozymes were applied at graded concentrations (200 to 0.78125 μg/ml) for 24 h, and viability was quantified using the Cell Counting Kit-8 (CCK-8) assay. Significant proliferation inhibition was observed at concentrations ≥25 μg/ml (Fig. S3), and 3.125 μg/ml was selected as the optimal working concentration and subsequently applied to all XPB formulations, at which no significant differences in cell viability were detected among groups (Fig. S4). Tube-formation analysis revealed that undoped PB had negligible effects on angiogenesis, whereas all doped variants exhibited enhanced tube formation to varying degrees (Fig. 2F to H). Notably, CuPB and Zn-doped PB (ZnPB) induced the most pronounced effects, increasing capillary branch points by 81.7% and 67.7%, and capillary lengths by 77.4% and 72.4%, respectively, compared with that of the blank control. Based on the superior performance in both antioxidant and angiogenic assays, CuPB was selected for subsequent mechanistic and therapeutic evaluations.

Finally, we compared CuPB against a physical mixture of free Cu ions and PB (Cu+PB) to validate the necessity of doping. In the oxygen evolution assay, the mixture failed to augment H_2_O_2_ decomposition rates relative to that of PB, whereas CuPB exhibited robust catalytic enhancement, confirming the critical role of lattice integration. Furthermore, in H_2_O_2_-challenged HUVECs, the Cu+PB mixture failed to rescue cell viability. This lack of cytoprotection stems directly from the mixture’s inability to enhance CAT-like activity, thereby failing to ameliorate the oxidative microenvironment. In contrast, CuPB significantly restored cell viability, attributing its superior efficacy to the structural doping that boosts catalytic kinetics (Figs. S5 and S6). To definitively validate the role of Cu ions, we employed a chelation strategy using d-penicillamine (DPA). In the free-Cu-ion group, DPA completely abolished angiogenic activity, confirming that Cu is the driver of vascularization. In sharp contrast, DPA failed to significantly inhibit the tube-forming activity of CuPB (Fig. S7). This resistance to chelation suggests that the robust coordination within the CuPB lattice prevents the rapid stripping of copper ions, thereby enabling stable, sustained delivery superior to free ionic forms.

### Structural characterization, ROS-scavenging activity, and catalytic mechanisms of screened CuPB nanozymes

**Fig. 3.**
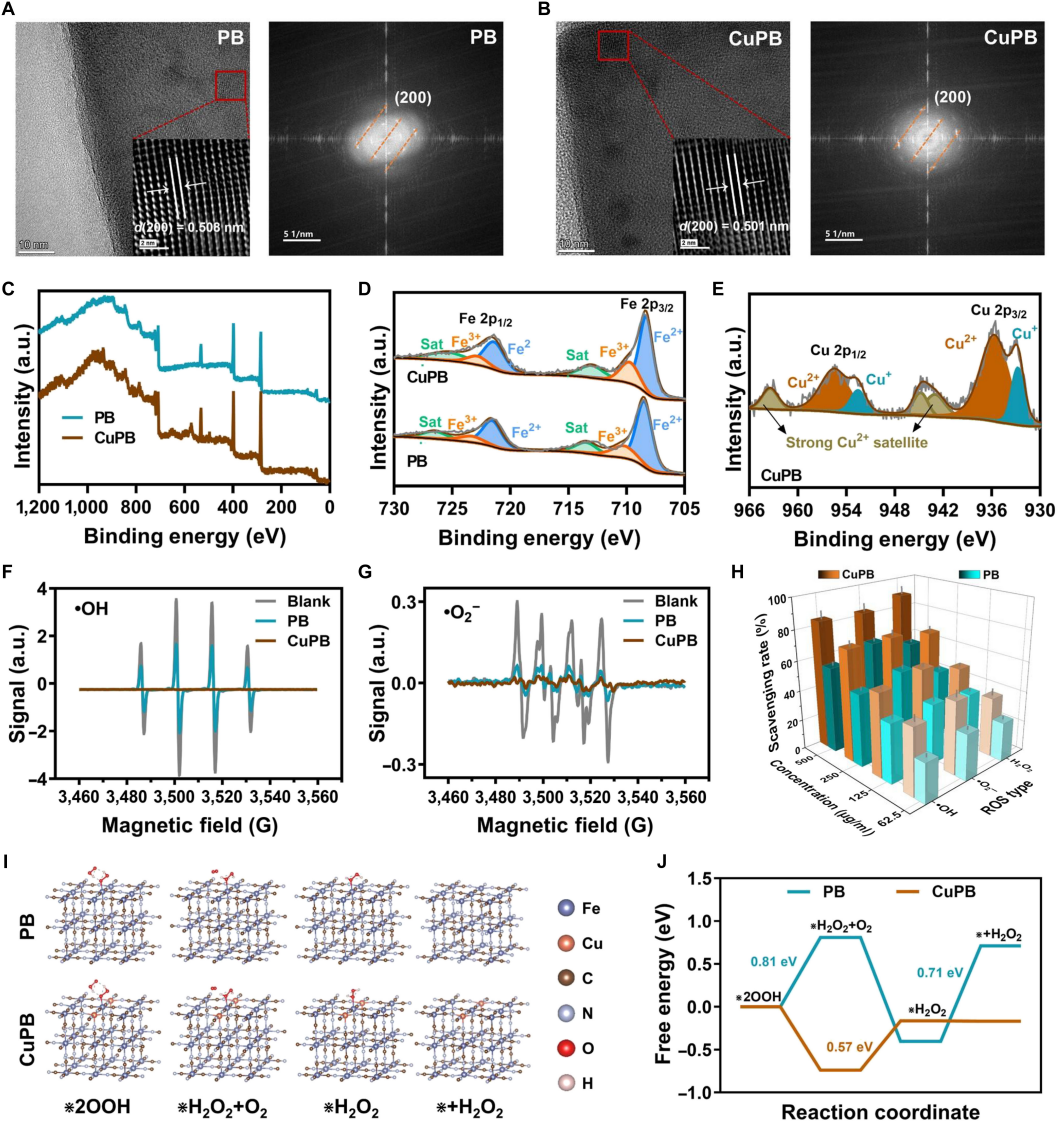
Structural characterization, reactive oxygen species (ROS)-scavenging activity, and catalytic mechanisms of screened Cu-doped Prussian blue (CuPB) nanozymes. (A and B) High-resolution transmission electron microscopy (HRTEM) images illustrating the lattice fringe spacing of Prussian blue (PB) and CuPB nanozymes. (C to E) X-ray photoelectron spectroscopy (XPS) spectra highlighting the characteristic Fe and Cu peaks in CuPB nanozymes. (F and G) Electron paramagnetic resonance (EPR) spectra assessing the scavenging capacity of PB and CuPB nanozymes against hydroxyl radicals (·OH) and superoxide anions (·O_2_^−^). (H) Quantitative assessment of the scavenging efficiencies for ·O_2_^−^, ·OH, and H_2_O_2_ by PB and CuPB nanozymes using ROS-specific assay kits. (I) Optimized molecular configurations from density functional theory (DFT) calculations of PB and CuPB nanozymes in the superoxide dismutase (SOD)-like reaction process. (J) Free-energy profiles derived from DFT calculations comparing the electron-transfer processes in PB and CuPB nanozymes.

The crystalline phases of PB and CuPB were first examined by x-ray diffraction. No additional diffraction peaks were detected after Cu doping, confirming that the incorporation of Cu did not alter the original PB phase (PDF: 52-1907, Fig. S8). High-resolution transmission electron microscopy further revealed a slight reduction in the (200) lattice spacing, from 0.508 nm in PB to 0.501 nm in CuPB (Fig. 3A and B). X-ray photoelectron spectroscopy analysis verified the successful incorporation of Cu, with the survey spectrum displaying distinct Cu signals. In the Cu 2p fine spectrum, peaks at 933.07 eV (Cu 2p_3/2_) and 952.87 eV (Cu 2p_1/2_) were accompanied by satellite features at 944.06 and 963.46 eV, consistent with Cu^2+^ as the predominant oxidation state and minor Cu^+^ species likely arising from localized reduction during substitution (Fig. 3C to E). The ion release results show that the doped Cu ions can be continuously released from CuPB, with no significant effect on the release of Fe ions from PB (Fig. S9). Subsequently, the enzyme-mimetic activity and ROS-scavenging performance of PB and CuPB were systematically assessed. Both nanozymes exhibited concentration-dependent scavenging of DPPH and ABTS^+^· radicals, with CuPB consistently outperforming PB at equivalent concentrations (Figs. S10 and S11). Electron paramagnetic resonance spectroscopy confirmed that both nanozymes effectively quenched ·OH and ·O_2_^−^ radicals, with a more pronounced effect observed for CuPB (Fig. 3F and G). Commercial assay kits further demonstrated that both nanozymes catalyzed the decomposition of ·OH, ·O_2_^−^, and H_2_O_2_, and in a dose-dependent manner, with CuPB showing significantly higher catalytic rates (Fig. 3H). These results indicate that both PB and CuPB possess robust SOD- and CAT-like activities, while Cu doping markedly enhances ROS-scavenging efficiency.

To elucidate the mechanism underlying this enhancement, density functional theory (DFT) simulations were performed to model SOD-like catalytic pathways in PB and CuPB. In both systems, 2 hydroperoxyl radicals (HO_2_·) adsorbed onto the catalyst surface undergo disproportionation to generate adsorbed hydrogen peroxide (*H_2_O_2_) and oxygen (*O_2_), which are subsequently released as H_2_O_2_ and O_2_, respectively (Fig. 3J). In CuPB, HO_2_· adsorption was energetically favorable, with a negative Gibbs free energy, whereas PB required overcoming an energy barrier of 0.81 eV (Fig. 3K). Moreover, H_2_O_2_ desorption occurred without an energy barrier in CuPB but required 0.71 eV in PB. These findings indicate that Cu doping reduces the catalytic energy barriers for key steps, thereby accelerating ROS conversion kinetics. In summary, the structural, compositional, and computational analyses confirm that Cu incorporation optimizes the catalytic pathway, yielding significantly enhanced antioxidant performance.

### CuPB nanozymes protect C2C12 myoblasts and H9c2 cardiomyocytes from H_2_O_2_-induced oxidative injury

**Fig. 4.**
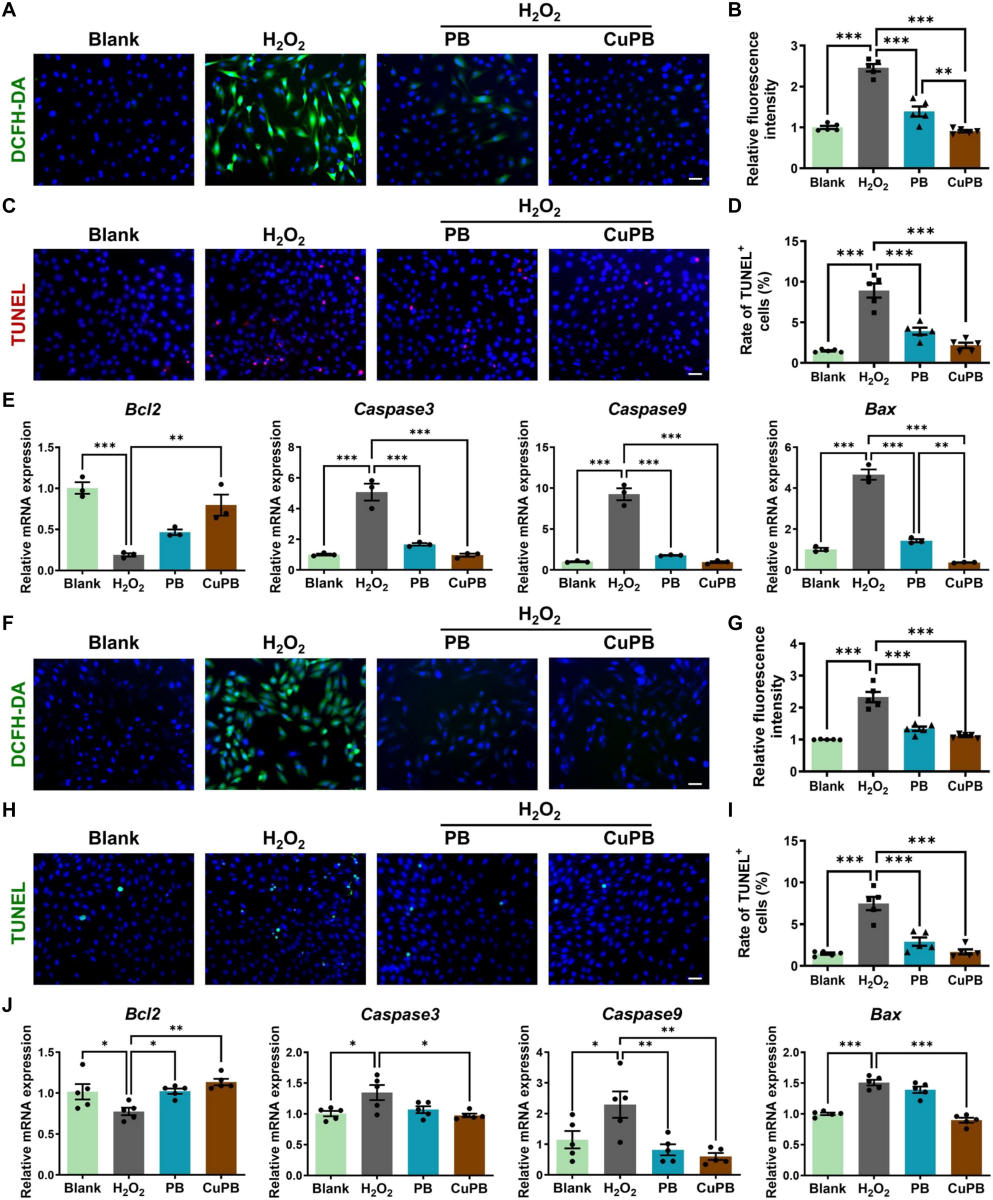
Cu-doped Prussian blue (CuPB) nanozymes protect C2C12 myoblasts and H9c2 cardiomyocytes from H_2_O_2_-induced oxidative injury. (A and B) Representative fluorescence images and quantification of intracellular reactive oxygen species (ROS) in H_2_O_2_-injured C2C12 cells after Prussian blue (PB) or CuPB treatment, detected using the 2′,7′-dichlorodihydrofluorescein diacetate (DCFH-DA) probe. Scale bar: 50 μm. *n* = 5. (C and D) Representative terminal deoxynucleotidyl transferase-mediated dUTP nick end labeling (TUNEL) staining images and quantification of apoptotic C2C12 cells following H_2_O_2_ injury with PB or CuPB treatment. Scale bar: 50 μm. *n* = 5. (E) Quantitative real-time polymerase chain reaction (qRT-PCR) analysis of apoptosis-related genes (*Bcl2*, *Caspase3*, *Caspase9*, and *Bax*) in C2C12 cells after different treatments. *n* = 3. (F and G) Representative fluorescence images and quantification of intracellular ROS in H_2_O_2_-injured H9c2 cells after PB or CuPB treatment, detected using the DCFH-DA probe. Scale bar: 50 μm. *n* = 5. (H and I) Representative TUNEL staining images and quantification of apoptotic H9c2 cells following H_2_O_2_ injury with PB or CuPB treatment. Scale bar: 50 μm. *n* = 5. (J) qRT-PCR analysis of apoptosis-related gene expression (*Bcl2*, *Caspase3*, *Caspase9*, and *Bax*) in H9c2 cells after different treatments. *n* = 5.

Given that muscle cells are among the most vulnerable somatic cell types in ischemic tissue injury, including limb ischemia and MI, 2 representative cell lines were selected: the C2C12 myoblast and the H9c2 cardiomyocyte lines. Oxidative-stress-induced injury was modeled in vitro by exposing C2C12 cells to 1,000 μM H_2_O_2_ for 4 h. This treatment significantly reduced cell viability compared with that of the blank control, whereas both PB and CuPB nanozymes markedly restored viability, with CuPB nanozymes achieving superior recovery (Fig. S12). Intracellular ROS levels, quantified using the 2′,7′-dichlorodihydrofluorescein diacetate (DCFH-DA) probe, were markedly elevated in H_2_O_2_-treated cells, as evidenced by intense green fluorescence. Treatment with PB or CuPB nanozymes significantly reduced fluorescence intensity, indicating effective ROS scavenging, with CuPB nanozymes again exhibiting a greater performance than PB nanozymes (Fig. 4A and B).

Oxidative stress may also lead to substantial apoptosis, as confirmed by terminal deoxynucleotidyl transferase-mediated dUTP nick end labeling (TUNEL) staining, which revealed a nearly 6-fold increase in TUNEL^+^ cells after H_2_O_2_ exposure compared with the blank control. PB and CuPB treatment reduced apoptosis to 43.79% and 24.23% of that of the H_2_O_2_ group, respectively, with CuPB providing stronger protection (Fig. 4C and D). Consistently, quantitative real-time polymerase chain reaction (qRT-PCR) analysis demonstrated that H_2_O_2_ injury up-regulated pro-apoptotic genes including *Caspase3* (cysteine-dependent aspartate-specific protease 3), *Caspase9*, and *Bax* by 3- to 8-fold and down-regulated the anti-apoptotic gene *Bcl2* (B-cell lymphoma 2) by >80%. Both PB and CuPB significantly reversed these changes, restoring expression toward baseline, with CuPB producing slightly greater modulation (Fig. 4E).

Comparable trends were observed in H9c2 cardiomyocytes. Exposure to H_2_O_2_ markedly reduced cell viability (Fig. S13) and increased intracellular ROS fluorescence compared with the blank control (Fig. 4F and G). Treatment with either PB or CuPB nanozymes restored cell viability and attenuated ROS accumulation, with CuPB consistently outperforming PB. TUNEL staining further revealed that CuPB nanozymes more effectively mitigated oxidative-stress-induced apoptosis than PB nanozymes (Fig. 4H and I). Consistent with these observations, qRT-PCR analysis demonstrated that CuPB more efficiently restored *Bcl2* expression and suppressed the expression of *Caspase3*, *Caspase9*, and *Bax* compared with PB (Fig. 4J).

Overall, these results demonstrate that CuPB nanozymes exhibit superior antioxidant and anti-apoptotic activity compared with PB, effectively alleviating oxidative-stress-induced injury in both skeletal and cardiac muscle cell models.

### CuPB nanozymes promote angiogenesis in HUVECs under both normal and oxidative stress conditions

**Fig. 5.**
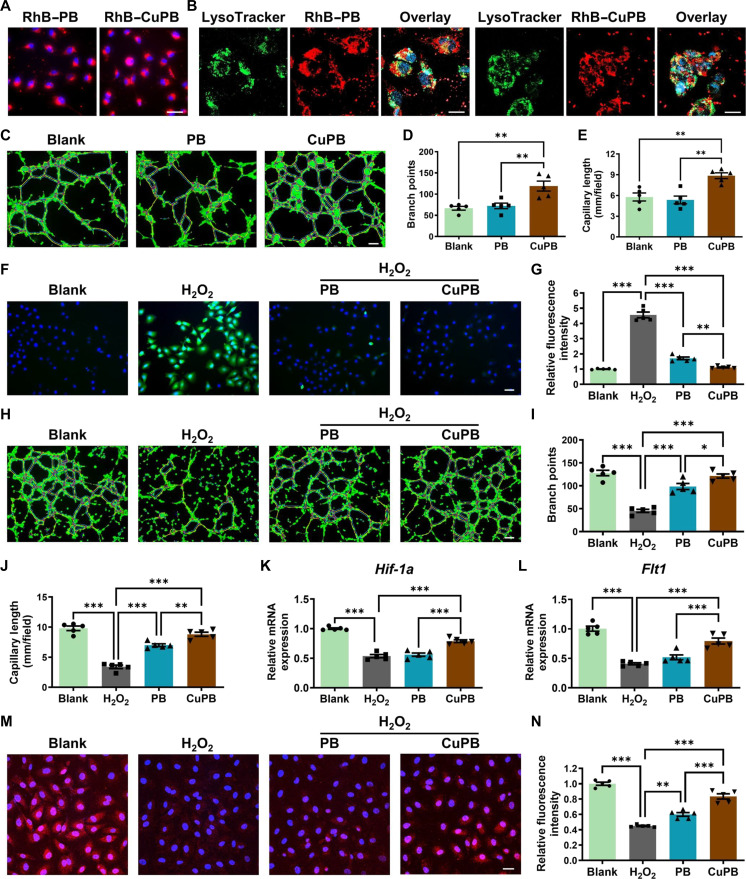
Cu-doped Prussian blue (CuPB) nanozymes promote angiogenesis in human umbilical vein endothelial cells (HUVECs) under both normal and oxidative stress conditions. (A) Cellular uptake of rhodamine B (RhB)-labeled Prussian blue (PB) or CuPB nanozymes (red) in HUVECs. Scale bar: 50 μm. (B) Colocalization of PB or CuPB nanozymes (red) with lysosomes (green) in HUVECs. Scale bar: 20 μm. (C to E) Representative images and quantification of vessel-like tube formation in HUVECs under normal conditions following treatment with PB or CuPB nanozymes. Scale bar: 100 μm. *n* = 5. (F and G) Representative fluorescence images and quantification of intracellular reactive oxygen species (ROS) in H_2_O_2_-injured HUVECs after PB or CuPB treatment, detected using the 2′,7′-dichlorodihydrofluorescein diacetate (DCFH-DA) probe. Scale bar: 50 μm. *n* = 5. (H to J) Representative images and quantification of vessel-like tube formation in H_2_O_2_-injured HUVECs following PB or CuPB treatment. Scale bar: 100 μm. *n* = 5. (K and L) Quantitative real-time polymerase chain reaction (qRT-PCR) analysis of angiogenesis-related gene expression (*Hif-1α* and *Flt1*) in H_2_O_2_-injured HUVECs treated with PB or CuPB nanozymes. *n* = 5. (M and N) Hypoxia-inducible factor 1-alpha (HIF-1α) immunofluorescence staining and quantitative assessment of the HIF-1α protein in H_2_O_2_-injured HUVECs after PB or CuPB treatment. Scale bar: 25 μm. *n* = 5.

In the initial screening, CuPB nanozymes demonstrated superior in vitro angiogenic potential compared with other formulations. Here, their angiogenic performance was characterized in greater detail. To assess intracellular delivery, HUVECs were incubated with rhodamine B-labeled PB or CuPB nanozymes for 4 h, resulting in distinct red fluorescence signals, confirming efficient cellular uptake (Fig. 5A). Colocalization analysis with LysoTracker (green) revealed extensive yellow signals in merged images, indicating that both PB and CuPB underwent endocytic uptake and accumulated within lysosomes, with no marked differences in trafficking patterns (Fig. 5B). The tube-forming ability of HUVECs was then examined by cytoskeletal staining. PB treatment had no appreciable effect on tube formation, whereas CuPB significantly increased the average number of capillary branches from 33.6 (blank) to 65.8 and the total capillary length from 2.44 ± 0.53 mm (blank) to 4.26 ± 0.53 mm (Fig. 5C to E). qRT-PCR analysis further revealed that CuPB, but not PB, significantly up-regulated the expression of proangiogenic genes *Hif-1α* and *Flt1* in HUVECs (Fig. S14), demonstrating the proangiogenic enhancement of Cu doping in PB nanozymes. Critically, the dependency of this process on *Hif-1α* was confirmed using the specific inhibitor lificiguat (YC-1); inhibition of *Hif-1α* effectively abrogated the angiogenic benefits of CuPB, identifying *Hif-1α* as a key mediator in CuPB-driven vascularization (Fig. S15).

Given that oxidative stress impairs angiogenesis, an H_2_O_2_-injured HUVEC model was established to mimic oxidative-stress-induced deficits in endothelial tube formation. DCFH-DA staining demonstrated markedly elevated ROS levels in H_2_O_2_-treated cells compared with those in the blank control. Both PB and CuPB significantly reduced ROS fluorescence intensity versus H_2_O_2_ alone, with CuPB exerting a more pronounced effect (Fig. 5F and G). Tube-formation assays showed that H_2_O_2_ treatment reduced branch points from 123.6 (blank) to 56.0, whereas PB and CuPB treatment restored them to 82.8 and 111.0, respectively. Correspondingly, the total capillary length increased from 3.39 ± 0.63 mm (H_2_O_2_) to 6.99 ± 0.51 mm (PB) and 8.80 ± 0.76 mm (CuPB), with CuPB again showing superior efficacy (Fig. 5H and I). Gene expression analysis revealed that H_2_O_2_ injury reduced *Hif-1α* and *Flt1* (VEGFR1 [vascular endothelial growth factor receptor 1]) transcript levels by ~50% relative to those in the blank controls (Fig. 5J to L). CuPB treatment significantly restored both genes toward baseline, outperforming PB. Immunofluorescence staining of HIF-1α corroborated these findings: oxidative injury lowered HIF-1α signal intensity, which was significantly recovered by both nanozyme treatments, with CuPB inducing a greater restoration than PB (Fig. 5M and N).

Taken together, these findings demonstrate that CuPB nanozymes not only enhance angiogenic gene expression and tube formation under physiological conditions but also more effectively reverse oxidative-stress-induced impairment of angiogenesis compared with undoped PB.

### Transcriptomic profiling of CuPB nanozyme-mediated angiogenic regulation in H_2_O_2_-injured HUVECs

Transcriptomic sequencing was performed to elucidate the molecular mechanisms underlying CuPB-mediated angiogenesis in HUVECs. Four groups were analyzed: blank control (blank), H_2_O_2_ injury (H_2_O_2_), H_2_O_2_ injury with PB-treated (PB), and H_2_O_2_ injury with CuPB-treated (CuPB). Principal component analysis revealed distinct transcriptional clustering among all groups, with the CuPB group positioned closer to the blank group than to the H_2_O_2_ group, indicating a more complete restoration of the baseline transcriptome (Fig. S16).

**Fig. 6.**
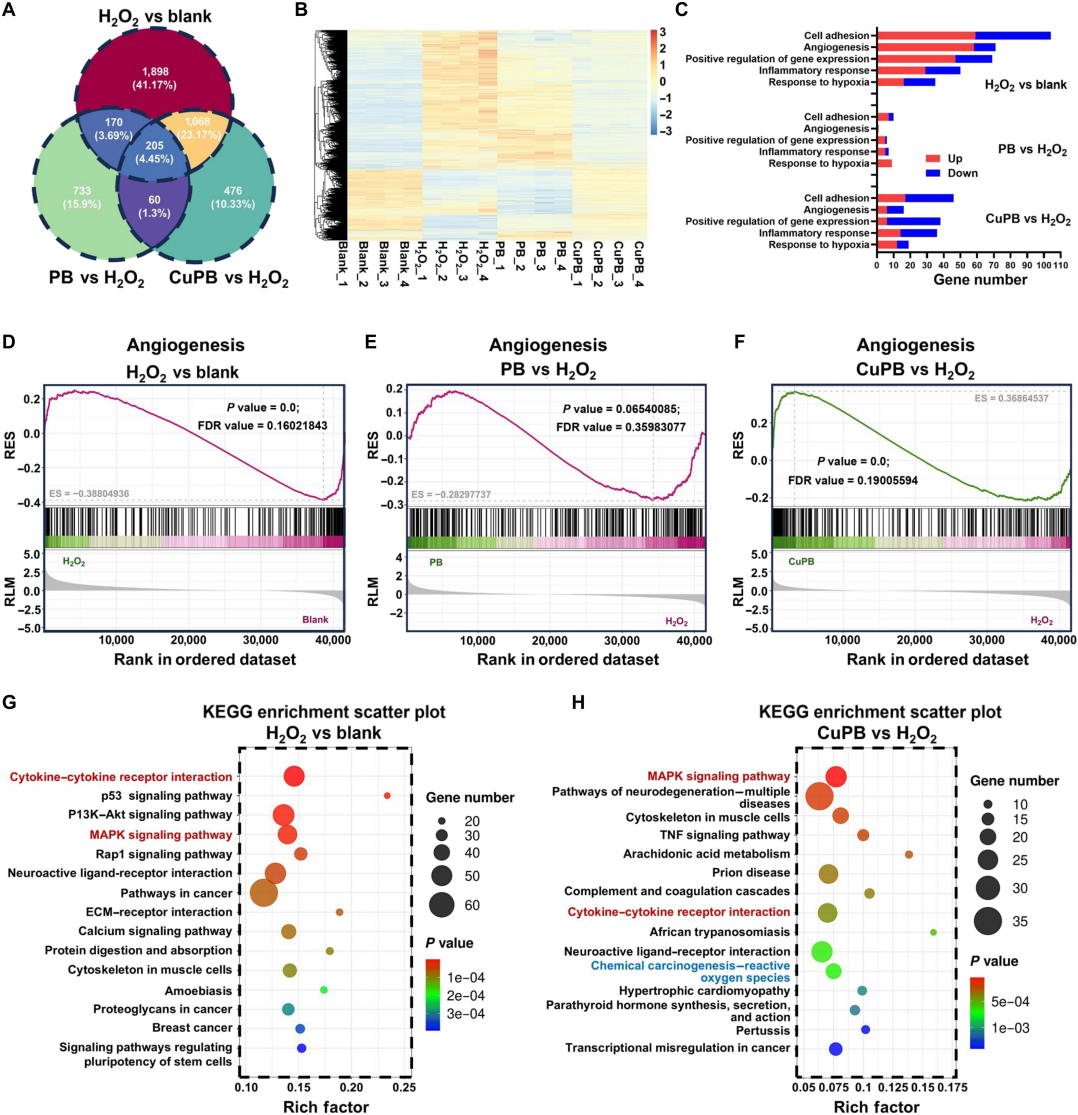
Transcriptomic profiling of Cu-doped Prussian blue (CuPB)-mediated angiogenic regulation in H_2_O_2_-injured human umbilical vein endothelial cells (HUVECs). (A) Venn diagram illustrating the overlap of differentially expressed genes (DEGs) among 3 pairwise comparisons: H_2_O_2_ vs blank, Prussian blue (PB) vs H_2_O_2_, and CuPB vs H_2_O_2_. (B) Heatmap showing hierarchical clustering of DEGs across the blank, H_2_O_2_, PB, and CuPB groups. (C) Top 5 Gene Ontology (GO) enrichment of biological processes altered by H_2_O_2_ and restored by PB or CuPB nanozymes. (D to F) Comparison of the GO term “angiogenesis” in (D) H_2_O_2_ vs blank, (E) PB vs H_2_O_2_, and (F) CuPB vs H_2_O_2_. (G) Top 15 Kyoto Encyclopedia of Genes and Genomes (KEGG) pathways enriched in DEGs between the H_2_O_2_ treatment group and the blank group (H_2_O_2_ vs blank). (H) Top 15 KEGG pathways enriched in DEGs between the CuPB treatment group and the H_2_O_2_ treatment group (CuPB vs H_2_O_2_).

Differential expression analysis showed that, relative to blank, H_2_O_2_ injury up-regulated 2,683 genes and down-regulated 658 genes. Compared with H_2_O_2_, PB treatment altered 861 genes upward and 307 downward, whereas CuPB treatment altered 386 genes upward and 1,423 downward (Fig. S17). Venn diagram analysis demonstrated that the overlap between H_2_O_2_ vs blank and PB vs H_2_O_2_ contained only 375 genes, whereas the overlap between H_2_O_2_ vs blank and CuPB vs H_2_O_2_ contained 1,273 genes, suggesting that CuPB reversed a substantially greater proportion of H_2_O_2_-induced transcriptional changes (Fig. 6A). Heatmap visualization further confirmed that the CuPB group displayed a transcriptomic profile more closely aligned with blank than the PB group (Fig. 6B). Gene Ontology enrichment of differentially expressed genes (DEGs) after oxidative injury identified 5 biological processes with the most significant alterations: “cell adhesion”, “angiogenesis”, “positive regulation of gene expression”, “inflammatory response”, and “response to hypoxia” (Fig. S18). Comparative analysis of gene expression in these categories revealed that CuPB elicited a more pronounced reversal of H_2_O_2_-induced dysregulation than PB (Fig. 6C). Gene set enrichment analysis further indicated that H_2_O_2_ injury suppressed angiogenesis-related signatures, whereas CuPB treatment restored them toward baseline; PB treatment failed to produce a statistically significant reversal (Fig. 6D to F). Similar restorative trends were observed for “response to hypoxia” and “inflammatory response” gene sets (Figs. S19 and S20).

Kyoto Encyclopedia of Genes and Genomes (KEGG) pathway analysis of the top 15 enriched pathways highlighted several signaling cascades implicated in angiogenic regulation. Notably, “cytokine–cytokine receptor interaction” and “MAPK signaling pathway” (highlighted in red) were significantly enriched in both H_2_O_2_ vs blank and CuPB vs H_2_O_2_ comparisons, suggesting their involvement in CuPB-mediated angiogenic restoration (Fig. 6G and H and Fig. S21). The “chemical carcinogenesis—reactive oxygen species” pathway (highlighted in blue) was enriched in both PB vs H_2_O_2_ and CuPB vs H_2_O_2_ groups, corroborating the potent antioxidant properties of these nanozymes.

Overall, both PB and CuPB partially reversed oxidative-stress-induced endothelial dysfunction and impaired angiogenesis. However, the restorative effect of CuPB was markedly greater, underscoring that antioxidant capacity alone is insufficient for full functional recovery. Instead, synergistic integration of ROS scavenging with proangiogenic modulation is essential for effectively restoring endothelial angiogenic function in oxidative stress environments.

### CuPB nanozymes restore perfusion and promote muscle regeneration in a mouse CLI model

**Fig. 7.**
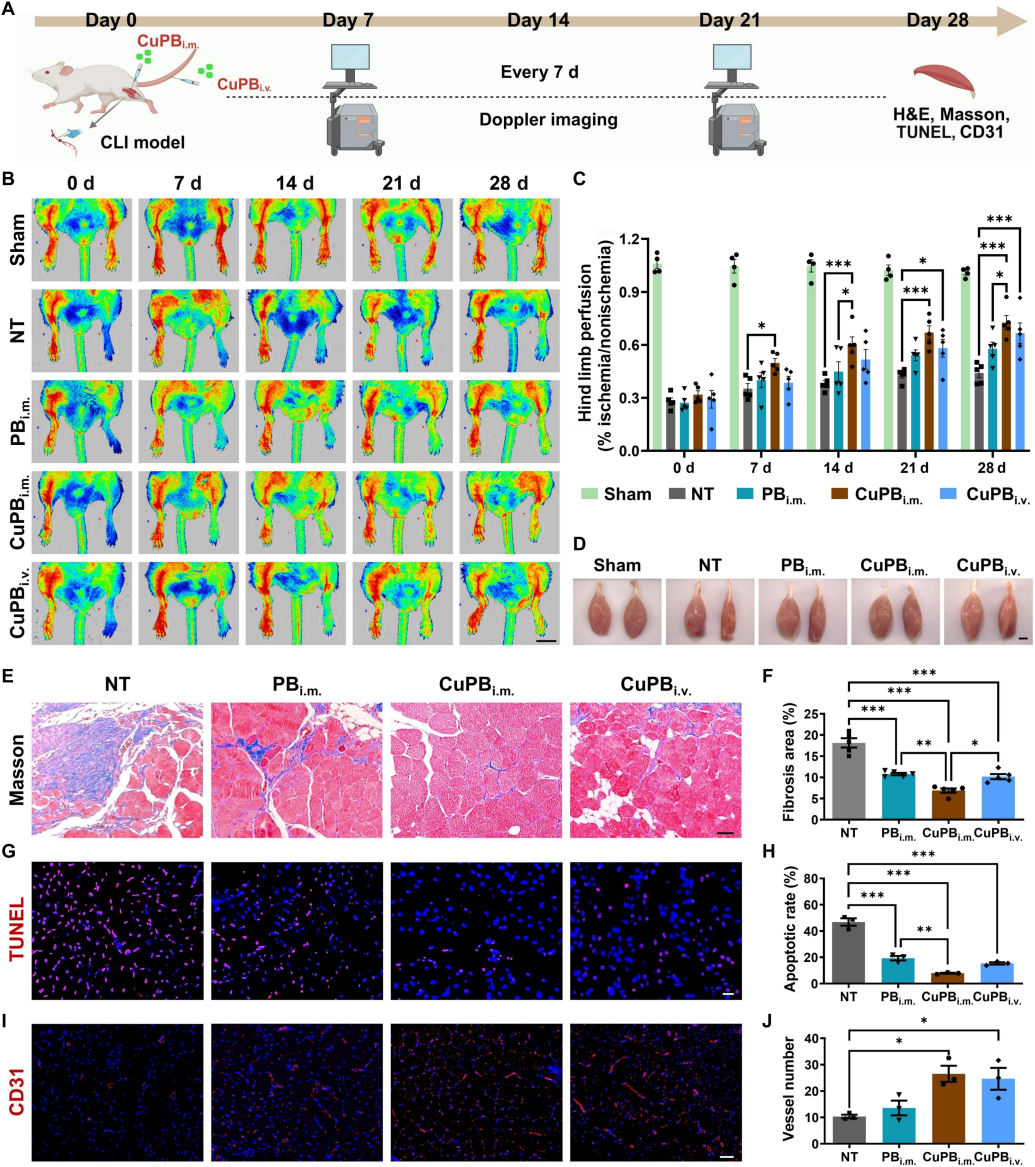
Cu-doped Prussian blue (CuPB) nanozymes restore perfusion and promote skeletal muscle regeneration after critical limb ischemia (CLI). (A) Schematic illustration of the experimental design. (B and C) Representative laser Doppler perfusion images of ischemic hind limbs after different nanozyme treatments and corresponding quantification of the ischemic-to-contralateral blood flow ratio. *n* = 4 for sham and *n* = 5 for others. Scale bar: 10 mm. (D) Gross morphology of gastrocnemius muscles on day 28 posttreatment. Scale bar: 250 μm. (E and F) Representative Masson’s trichrome staining sections and quantification of fibrosis in gastrocnemius muscle. Scale bar: 40 μm. *n* = 5. (G and H) Representative terminal deoxynucleotidyl transferase-mediated dUTP nick end labeling (TUNEL)-stained sections and quantification of apoptotic cell percentage in gastrocnemius muscle. Scale bar: 25 μm. *n* = 3. (I and J) Representative immunofluorescence images of CD31 and quantification of microvessel density in gastrocnemius muscles. Scale bar: 50 μm. *n* = 3. (A) was created with BioRender.com.

To evaluate the therapeutic potential of PB and CuPB nanozymes, a mouse CLI model was established by double ligation of the femoral artery. Nanozymes were administered either via intramuscular injection at 4 distinct hind limb sites (PB_i.m._ and CuPB_i.m._) or via intravenous tail-vein injection (CuPB_i.v._) (Fig. 7A). An administration dose of 10 mg/kg was selected based on the established literature, which defines 5 to 20 mg/kg as the conventional therapeutic and safety window for standard PB nanozymes in murine models of ischemia and inflammation [30–32]. Laser Doppler perfusion imaging confirmed complete loss of hind limb blood flow immediately after surgery in all groups (Fig. 7B). By day 14, the CuPB_i.m._ group exhibited marked restoration of perfusion, whereas the PB_i.m._ and CuPB_i.v._ groups showed only modest improvement, and untreated (NT) mice showed persistent ischemia even at day 28. Quantitative analysis revealed that the perfusion ratio (ischemic/contralateral limb) in the CuPB_i.m._ group reached 0.48 ± 0.06 on day 7, 0.61 ± 0.10 on day 14, 0.67 ± 0.08 on day 21, and 0.72 ± 0.08 on day 28, approaching the baseline levels of the contralateral limb (Fig. 7C). Recovery in the PB_i.m._ and CuPB_i.v._ groups was markedly inferior with heterogeneous perfusion patterns. These findings indicate that local delivery of CuPB nanozymes significantly accelerates and sustains blood flow restoration in ischemic limbs.

On day 28, gastrocnemius muscles were collected for morphological and histological evaluation. Gross inspection revealed severe atrophy in the NT group, partial preservation in the PB_i.m._ and CuPB_i.v._ groups, and near-normal morphology in CuPB_i.m._, closely resembling that in sham controls (Fig. 7D). Wet weight analysis confirmed that muscle mass in CuPB_i.m._ was statistically indistinguishable from that in sham and significantly greater than those in all other treatment groups (Fig. S22). Histological evaluation via hematoxylin–eosin (H&E) and Masson’s trichrome staining showed extensive myofiber degeneration and collagen deposition in NT mice, partially alleviated by PB_i.m._ and CuPB_i.v._ but almost completely prevented in CuPB_i.m._ (Fig. 7E and F and Fig. S23). Consequently, TUNEL staining was conducted for the assessment of muscle cell apoptosis, which demonstrated significant apoptosis in NT muscles, reduced by all treatments, with CuPB_i.m._ showing the most pronounced anti-apoptotic effect (Fig. 7G and H). To further validate the mitigation of oxidative-stress-induced injury in vivo, we assessed the expression of cleaved caspase-3, a key executor of apoptosis triggered by ROS. Immunohistochemical analysis revealed that CuPB treatment significantly suppressed cleaved caspase-3 expression in ischemic muscle tissue compared to that in the NT group (Fig. S24). This finding aligns with the TUNEL staining results, confirming that CuPB effectively protects cells from oxidative-damage-mediated apoptosis. CD31 immunofluorescence staining was used to evaluate angiogenesis. Both CuPB_i.m._ and CuPB_i.v._ markedly increased neovascularization, with CuPB_i.m._ producing the highest microvessel density, whereas PB_i.m._ induced only a modest, nonsignificant increase (Fig. 7I and J). In addition, systemic biosafety evaluation via H&E staining of major organs (heart, liver, spleen, lung, and kidney) revealed no observable histopathological abnormalities in any treatment group, confirming the biocompatibility of both PB and CuPB nanozymes (Fig. S25).

We further assessed cellular internalization and tissue residence time using cyanine 5.5 (Cy5.5)-labeled nanozymes. ICP-MS quantification indicated comparable cellular uptake efficiencies for PB and CuPB (~0.6%, Fig. S26), with no significant differences observed. This trend was mirrored in vivo; fluorescence imaging of the ischemic hind limb confirmed that both formulations achieved robust local retention. The signal persisted for over 14 d postinjection, with both PB and CuPB exhibiting nearly identical decay kinetics (Fig. S27).

By day 14, low-intensity fluorescence was detected in the liver and spleen, suggesting clearance through the reticuloendothelial and renal pathways. Fluorescence was also observed in kidney tissue, likely associated with renal excretion of free probes or degradation products. In contrast, intravenously injected CuPB rapidly distributed to the heart, liver, kidney, and ischemic muscle within 6 h, with markedly reduced signal in all tissues by 24 h, indicating efficient systemic clearance (Fig. S28).

Collectively, these results demonstrate that intramuscular delivery of CuPB nanozymes provides superior outcomes, including restoration of perfusion, preservation of muscle integrity, inhibition of apoptosis, and stimulation of neovascularization, compared with intramuscular delivery of PB or systemic delivery of CuPB, highlighting their translational potential for ischemic limb repair.

### CuPB nanozymes enhance cardiac function and promote myocardial regeneration in a mouse MI model

**Fig. 8.**
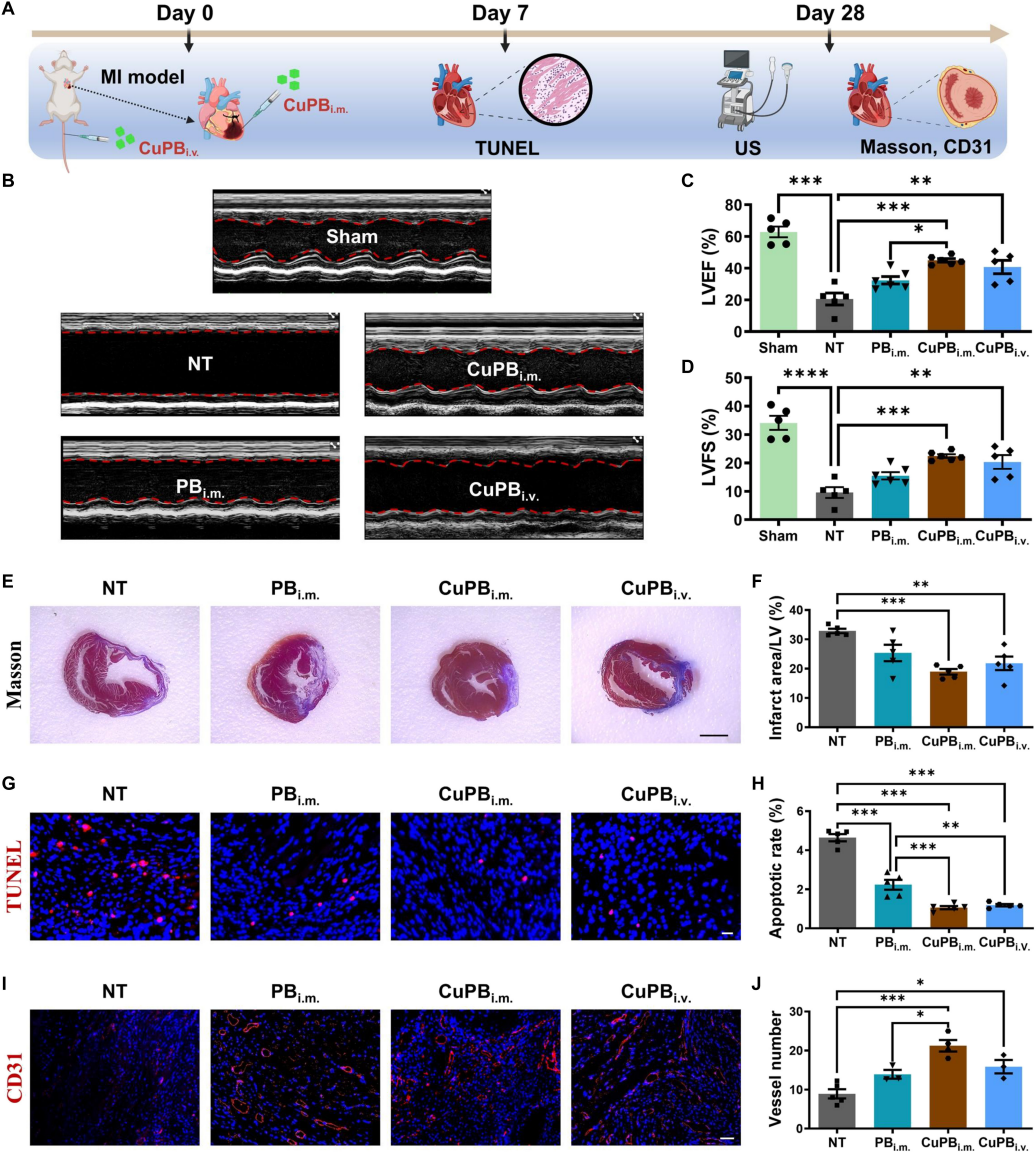
Cu-doped Prussian blue (CuPB) nanozymes enhance cardiac function and promote myocardial regeneration after myocardial infarction (MI). (A) Schematic illustration of the experimental design. (B to D) Representative M-mode echocardiogram images and quantification of left ventricular ejection fraction (LVEF) and left ventricular fractional shortening (LVFS) on day 28 post-MI in mice receiving different nanozyme treatments. *n* = 5. (E and F) Representative Masson’s trichrome staining images and quantification of fibrosis in the infarct border zone. Scale bar: 2 mm. *n* = 5. (G and H) Representative terminal deoxynucleotidyl transferase-mediated dUTP nick end labeling (TUNEL) staining images and quantification of apoptotic cell percentage in the infarct border zone. Scale bar: 25 μm. *n* = 5. (I and J) Representative immunofluorescence images of CD31 and quantification of microvessel density in the infarct border zone. Scale bar: 50 μm. *n* = 3. (A) was created with BioRender.com.

Besides the ischemic limb model, a mouse MI model was established to assess the therapeutic effects of CuPB nanozymes. The MI model was generated by ligation of the left anterior descending coronary artery (LAD), followed by intramyocardial injection of nanozymes at peri-infarct sites (PB_i.m._ and CuPB_i.m_.) or intravenous tail-vein injection (CuPB_i.v._) (Fig. 8A). On day 28, cardiac function was evaluated using high-resolution echocardiography (Fig. 8B). In NT mice, the left ventricular ejection fraction (LVEF) fell sharply from 62.91% ± 7.64% (sham) to 20.61% ± 8.49%, and the left ventricular fractional shortening (LVFS) dropped from 34.11% ± 5.54% to 9.57% ± 4.20%. CuPB_i.m._ significantly improved LVEF to 44.98% ± 2.73% and LVFS to 22.42% ± 1.54%, outperforming CuPB_i.v._ (LVEF 40.76% ± 9.54%; LVFS 20.34% ± 5.39%) and PB_i.m._ (LVEF 32.76% ± 5.79%; LVFS 15.46% ± 3.08%) (Fig. 8C and D). These results indicate that in situ injection of CuPB is more effective than PB or tail-vein injection of CuPB in protecting and restoring cardiac function.

The extent of cardiac fibrosis was then evaluated by Masson’s trichrome staining, where fibrotic regions appeared blue, and normal myocardium, red (Fig. 8E and F). The NT group displayed a fibrosis ratio of 32.86% ± 1.67%. Both CuPB_i.m._ and CuPB_i.v._ treatments significantly reduced fibrosis, with CuPB_i.m._ achieving the most pronounced decrease to 18.96% ± 2.14%. Although CuPB_i.v._ also reduced fibrosis compared with NT, the decrease did not reach statistical significance. Myocardial apoptosis at day 7 was assessed by TUNEL staining, which revealed that all treatments significantly reduced the myocardial apoptosis rate compared with that of the NT group. Specifically, apoptosis in NT mice (4.64% ± 0.41%) was reduced to 1.06% ± 0.18% in the CuPB_i.m._ group and 1.18% ± 0.13% in the CuPB_i.v._ group, both of which were superior to that of PB_i.m._ treatment (2.23% ± 0.56%) (Fig. 8G and H). In contrast to the early apoptotic assessment, CD31 immunofluorescence staining on myocardial tissue collected 28 d post-MI revealed distinct angiogenic patterns. While the PB_i.m._ group showed a higher number of vessels than the NT group, the difference was not statistically significant. In comparison, both local and systemic administration of CuPB nanozymes significantly increased microvessel density in peri-infarct regions, with CuPB_i.m._ producing a more pronounced proangiogenic effect than CuPB_i.v._ (Fig. 8I and J). Von Willebrand factor and alpha smooth muscle actin staining also observed that CuPB_i.m._ significantly promoted vascular maturation within the ischemic myocardium, suggesting the formation of stable, functional vessels (Fig. S29). Furthermore, systemic safety evaluation via H&E staining of major organs (liver, spleen, lung, and kidney) revealed no pathological alterations in any group, confirming the favorable biosafety profile of both PB and CuPB nanozymes (Fig. S30).

Together, these findings demonstrate that CuPB nanozyme administration confers superior cardiomyocyte protection, promotes neovascularization, and thereby mitigates fibrosis and preserves cardiac function, underscoring their translational potential for MI therapy.

## Discussion

During tissue ischemia, the blood supply is disrupted, markedly reducing the delivery of oxygen and nutrients to the affected region. This reduction impairs adenosine triphosphate production via oxidative phosphorylation, forcing cells to switch metabolic pathways and compromising energy-dependent functions [33]. The reduced availability of adenosine triphosphate imposes limitations on the ion pumps on the cell membrane, triggering calcium overload and cellular structural disarray, ultimately culminating in necroptosis of the cells [34]. These dying cells further induce excessive oxidative stress and continuously secrete ROS, which in turn triggers an inflammatory storm and accelerates the process of secondary cell necrosis [35,36]. To address this issue, ROS scavenging has been widely recognized as an effective strategy to mitigate oxidative stress, suppress inflammatory cascades, and limit secondary injury. Among the current ROS-scavenging biomaterials, nanozymes have attracted considerable attention due to their capabilities to precisely eliminate ROS by mimicking the functions of SOD and CAT [16,37]. However, conventional nanozymes generally lack intrinsic biological activities to stimulate tissue regeneration. In ischemic diseases, such catalytic activity alone cannot activate endothelial cells in hypoxic regions or initiate angiogenesis, thus precluding complete tissue repair. Although strategies such as protein functionalization, cell membrane coating, and polymer conjugation have been explored to confer bioactivity [38], these surface modifications introduce exogenous agents that not only increase the complexity of the material but may also impair catalytic accessibility and lead to loss of functionality upon degradation. Consequently, engineering nanozymes with inherent bioactivity, achieved via material design rather than surface ornamentation, is key to overcoming this therapeutic bottleneck.

To tackle this challenge, the study proposes an ion-doping strategy, modifying traditional PB nanozymes with proangiogenic elements (Mg, Co, Cu, Zn, Sr, and Eu). Systematic screening identified Cu doping as the most effective modification, endowing PB with a strong angiogenic activity and enabling therapeutic efficacy in murine models of hind limb ischemia (HI) and MI. This approach provides a generalizable strategy for directly integrating functional inorganic elements into nanozymes to impart targeted bioactivities, such as angiogenesis, immune modulation, or anti-inflammation, without compromising catalytic performance. In this study, the proangiogenic function of CuPB is likely attributed to the presence of Cu ions. Previous studies have shown that Cu ions play a crucial role as activators in the HIF/VEGF/VEGFR2 signaling pathway, which is a well-known and essential proangiogenic mechanism [39]. Functioning as an intracellular delivery vehicle, CuPB releases bioactive copper ions to trigger this pathway. This hypothesis was substantiated by our inhibitor studies: treatment with YC-1, a specific HIF-1α inhibitor, significantly attenuated the angiogenic capacity of CuPB, confirming that the observed vascularization is indeed driven by copper-mediated HIF-1α activation and subsequent VEGF/VEGFR2 signaling. Expanding upon this mechanism, transcriptome profiling revealed the concurrent activation of additional angiogenesis-related networks, such as cytokine–cytokine receptor interactions and the MAPK signaling pathway. These findings suggest that CuPB operates through the synergistic orchestration of multiple mechanisms, offering a multipronged therapeutic strategy to accelerate tissue repair and revascularization in ischemic microenvironments.

Intriguingly, CuPB nanozymes not only acquire proangiogenic activity but also significantly enhance the intrinsic catalytic performance of PB. DFT calculations revealed that the (001) crystal plane of CuPB exhibits a significantly lower energy barrier during the electron-transfer process compared to PB, which must overcome a much higher energy barrier to achieve catalysis. This difference directly elucidates the reason for the substantial improvement in the catalytic activity of CuPB. Further analysis revealed that Cu doping induces subtle structural adjustments in PB, which may lead to the formation of local structural defects. Previous studies have demonstrated that elemental doping can cause lattice distortion or defects in nanozyme materials, thereby generating more active sites, optimizing the electronic structure, and regulating the energy-band distribution, ultimately enhancing the adsorption capacity of reactants and the efficiency of electron transfer [40,41]. This structural optimization may enable CuPB to more efficiently come into contact with ROS and undergo catalytic reactions [42,43]. It is noteworthy that Cu itself serves as a highly efficient catalytic active center [44]. For instance, in SOD, Cu^2+^ coordinates with the imidazole nitrogens of 4 histidine residues, forming a distorted tetrahedral structure. The redox cycling between Cu^2+^ and Cu^+^ drives the disproportionation of O_2_^−^ [44,45]. Similarly, Cu doping may form multimetallic active centers with metals such as Fe in PB, triggering a synergistic catalytic effect and thus significantly enhancing the overall catalytic activity.

In ischemic disease therapy, uncontrolled promotion of angiogenesis may lead to adverse outcomes. For example, in myocardial ischemia, experimental data show that reperfusion causes a several-fold surge in ROS production [46,47]. This surge occurs because reperfusion reintroduces oxygen into the tissue, overwhelming the mitochondrial electron transport chain due to accumulated nicotinamide adenine dinucleotide/nicotinamide adenine dinucleotide (NADH/FADH_2_), leading to electron leakage and massive O_2_^−^ production. This superoxide is further converted to H_2_O_2_ or ·OH via SOD and Fenton reactions, markedly increasing ROS levels during reperfusion. A similar mechanism operates in lower limb ischemia. Skeletal muscle contains relatively high levels of xanthine dehydrogenase, which is converted to xanthine oxidase during ischemia through calcium overload or protease activation. After reperfusion, xanthine oxidase utilizes oxygen to oxidize hypoxanthine into uric acid, releasing a large amount of ·O_2_^−^ and H_2_O_2_ in the process, thereby further aggravating oxidative stress [48,49]. This paradoxical phenomenon highlights the limitations of conventional strategies: promoting angiogenesis alone restores perfusion but induces explosive ROS generation that exacerbates injury, whereas failing to restore blood supply accelerates apoptosis and necrosis due to energy depletion. This dilemma underlies the limited efficacy of proangiogenic factors such as VEGF in treating ischemic diseases; simply inducing angiogenesis may provoke “therapeutic injury”.

The proangiogenic CuPB nanozymes designed in this study effectively resolve this contradiction through a dual-functional mechanism. On the one hand, CuPB can instantaneously and efficiently catalyze the decomposition of ROS, rapidly blocking the explosive generation of ROS after blood flow restoration and preventing secondary tissue damage caused by oxidative stress. On the other hand, its proangiogenic effect synergizes with its ROS-scavenging function, ensuring the reconstruction of blood supply while inhibiting oxidative stress, establishing a closed-loop mechanism of protection and repair. In mouse models of CLI and MI, the dual efficacy of CuPB markedly outperforms the single-function treatment provided by PB. The core advantage of CuPB resides in its capacity to promptly eliminate ROS during the early phase of blood flow restoration, which corresponds to the peak period of ROS generation. Meanwhile, the synergy between its proangiogenic and antioxidative functions maximizes the efficiency of tissue repair. The study offers a new perspective for the treatment of ischemic diseases, namely, achieving dynamic regulation of therapeutic targets through material design to balance blood supply reconstruction and oxidative stress inhibition, thereby breaking through the limitations of traditional therapies.

In the realm of research and treatment of ischemic diseases, a critical and often overlooked issue is that various types of ischemic diseases do not exist in isolation but are highly likely to occur concurrently, forming a complex network of vascular complications. For instance, approximately 60% of patients with PAD also suffer from IHD [50]. This is because vascular pathology often exhibits systemic characteristics, where lesions in one vascular bed may signal potential risks in others. For patients with IHD, arterial embolism occurring in the proximal heart is not confined to the local cardiac region; it can extend through the bloodstream to the extremities, triggering distal ischemic symptoms and further exacerbating the patient’s disease burden [51,52]. Another notable advantage of CuPB, as designed in this study, is its remarkable therapeutic efficacy in both distal ischemic models, exemplified by murine HI, and proximal ischemic models, represented by murine MI. This finding holds significant implications, suggesting that CuPB may possess the capacity to prevent the comorbidity of ischemic diseases. Specifically, when CuPB is administered during the treatment of IHD, it can readily distribute through the systemic circulation via circulation and exert its effects systemically, thereby effectively preventing distal ischemic diseases that may arise from the extension of myocardial pathology to the extremities and providing more comprehensive protection for patients. Similarly, in the treatment of patients with PAD, CuPB can also reach the myocardial region through the circulatory system, enabling early intervention to prevent the potential onset of IHD, thus achieving a transition from local treatment to systemic prevention. Remarkably, during the treatment of murine MI and HI with CuPB, no toxic effects were observed in any tissues or organs. This unique characteristic empowers CuPB to genuinely achieve systemic treatment through circulating blood, representing a significant clinical translation advantage of the proangiogenic nanozyme over ordinary nanozymes. Furthermore, given that other ischemic pathologies, such as ischemic stroke and renal ischemia, share key etiological hallmarks, specifically oxidative stress and hypoperfusion, it is theoretically plausible that CuPB exerts similar therapeutic efficacy across these conditions. Collectively, this work opens new horizons for the broad-spectrum management of ischemic diseases, promising safer and more efficacious therapeutic alternatives for patients.

Notably, this study investigated 2 distinct therapeutic paradigms: local administration, involving in situ injection of CuPB into the lesion (CuPB_i.m._), and systemic administration via tail-vein injection (CuPB_i.v._). A comparative analysis revealed that CuPB_i.m._ elicited superior therapeutic outcomes relative to those of CuPB_i.v._, characterized by enhanced antifibrotic, anti-apoptotic, and proangiogenic efficacy. This advantage is likely attributable to the ability of localized delivery to rapidly modulate the cellular microenvironment within the injured tissue, rendering CuPB_i.m._ particularly well suited for the intervention of acute ischemic conditions. Conversely, although CuPB_i.v._ showed lower immediate potency than its local counterpart, it still provided significant benefits over the NT group. Given the broad biodistribution facilitated by systemic circulation, CuPB_i.v._ holds potential for comprehensive management or the treatment of chronic, multifocal pathologies such as heart failure and atherosclerosis [53,54].

However, this study still has certain limitations. Firstly, during the construction of doping-engineered nanozymes with proangiogenic properties, this study subjectively selected a portion of representative bioactive ions. Based on the assessments of catalytic ability and angiogenic capacity, CuPB was chosen as the research subject. Nevertheless, in reality, a substantial number of bioactive ions have not been incorporated into the functional screening system. Consequently, it remains uncertain whether CuPB represents the optimal choice for constructing doping-engineered proangiogenic nanozymes at present. Furthermore, given the well-established synergistic effects among different metal elements [55], it is theoretically plausible that multielemental doping strategies could yield functionalities superior to the single-doped CuPB system. Consequently, future investigations should focus on the systematic exploration of combinatorial doping ratios and compositions to identify the definitive optimal formulation. Secondly, whether the currently employed construction scheme for doping-engineered proangiogenic nanozymes is applicable to nanozyme systems other than PB still requires further validation. In response to the aforementioned issues, subsequent research could introduce machine learning technology to carry out big data screening, aiming to optimize the doping schemes for different nanozymes [56–59]. Thirdly, regarding the mechanism of ion-doped nanozymes, this study primarily focuses on the lead candidate CuPB. Our screening data indicated that Cu and Zn exhibited the strongest angiogenic potential, which we attribute to their direct involvement as cofactors in HIF-1α and eNOS signaling, respectively. However, a comprehensive comparative analysis of the molecular signaling landscapes induced by the full spectrum of doped ions (Mg, Co, Sr, and Eu) was not performed. Future investigations should employ high-throughput transcriptomics or proteomics to systematically map the divergent signaling cascades elicited by different metal ions, thereby providing a deeper theoretical basis for ion-based material design. Additionally, differences in therapeutic efficacy were observed between local and systemic administration of CuPB, which may be attributed to variations in the administration routes. Finally, although CuPB administered at 10 mg/kg exhibited significant therapeutic efficacy without inducing observable histopathological alterations in major organs, a precise therapeutic dose window has not yet been delineated. The safety profile of conventional PB cannot be directly extrapolated to metal-doped nanozymes such as CuPB [30–32]. Consequently, the absence of empirically defined maximal effective doses and maximum tolerated doses constitutes a limitation of this study. For future clinical translation, it is imperative to conduct comprehensive, glucagon-like peptide (GLP)-compliant, dose-escalation pharmacokinetic and toxicological evaluations in large animal models. Such investigations are essential to precisely establish the therapeutic index of this proangiogenic nanozyme and to systematically assess long-term safety, particularly concerning chronic toxicity and the potential for aberrant neovascularization.

In summary, this study established a robust screening platform for angiogenic ion-doped nanozymes and successfully identified CuPB as the optimal formulation. Comprehensive in vivo evaluations in murine models of 2 representative ischemic conditions, HI and MI, demonstrated that CuPB confers potent dual therapeutic benefits. On one hand, it rapidly and efficiently catalyzes ROS decomposition, thereby mitigating secondary tissue injury caused by oxidative stress. On the other hand, it actively stimulates rapid vascular regeneration, expediting blood flow restoration. Importantly, the proangiogenic effect of CuPB operates in synergy with its pronounced ROS-scavenging activity, effectively suppressing the surge of ROS commonly observed during reperfusion and preventing associated “therapeutic injury”. Collectively, these findings underscore an innovative material design strategy, incorporating proangiogenic inorganic elements directly into the nanozyme framework, to endow nanozymes with intrinsic vascular regenerative capacity. This approach offers a promising paradigm for the comprehensive treatment of ischemic diseases, addressing both oxidative damage control and perfusion recovery in a single therapeutic platform.

## Materials and Methods

### Materials

All chemicals were purchased from Macklin Biochemical Technology Co., Ltd. (Shanghai, China) unless otherwise specified.

### Synthesis of PB and angiogenic XPB nanozymes

A mixed solution containing 929.04 mg of potassium ferricyanide (K_3_[Fe(CN)_6_]), 24 g of polyvinylpyrrolidone (PVP; K30), and 320 ml of 0.01 M HCl was subjected to magnetic stirring for 30 min until it turned yellow and clear. Subsequently, the mixture was transferred to a 3-necked flask and heated to 80 °C while being stirred for 20 h, resulting in a green liquid. After being allowed to age at room temperature for 24 h, the precipitate was collected by centrifugation (12,000 rpm, 20 min) and washed 6 times with deionized water. Finally, the PB nanozyme was obtained through lyophilization.

XPB refers to angiogenic ions doped PB nanozymes, including CuPB, ZnPB, MgPB, CoPB, EuPB, and SrPB. Briefly, 50 mg of the previously synthesized PB, 67.5 mg of sodium citrate dihydrate (Na_3_C_6_H_5_O_7_·2H_2_O), and 250 mg of PVP (K30) were dissolved in 20 ml of deionized water, and this solution was labeled as solution A. Aqueous solutions of 0.1 mmol of Cu(CH_3_COO)_2_·H_2_O, Zn(CH_3_COO)_2_·2H_2_O, Mg(CH_3_COO)_2_, Co(CH_3_COO)_2_·4H_2_O, EuCl_3_·6H_2_O, or Sr(CH_3_COO)_2_ were labeled as solution B. Different solution B samples were separately mixed with solution A and stirred for 3 h. Then, 66 mg of K_3_[Fe(CN)_6_] was added, and the mixtures were continuously stirred for another 2 h. After that, the mixtures were allowed to age at approximately room temperature (25 °C) for 24 h. The supernatants were removed by centrifugation, and the precipitates were washed 6 times with deionized water. The XPB nanozymes were finally obtained by freeze-drying.

To ensure the synthesis of PB and XPB with uniform cubic morphologies, appropriate amounts of PVP and HCl were introduced. PVP acts as a capping agent that preferentially adsorbs onto specific facets of the PB nuclei, thereby guiding the formation of a cubic structure. Concurrently, HCl fosters a slow crystallization environment that suppresses rapid precipitation, allowing sufficient time for the precursors to organize into the thermodynamically favored cubic lattice configurations.

### Characterization of nanozymes

#### Morphological and structural characterization

The morphologies of the synthesized PB and XPB nanozymes were observed using a field-emission scanning electron microscope (Zeiss Sigma 300, Germany). The morphology and elemental distribution of the XPB powders were examined using a high-resolution transmission electron microscope equipped with an energy-dispersive x-ray spectrometer (Oxford Ultim Max 65, UK). The crystal structures of the samples were investigated using an x-ray diffractometer (Bruker AXS D8). The hydrodynamic diameters and zeta potentials of the materials were analyzed using a nanoparticle size analyzer (Malvern, Zetasizer Nano ZS90) in combination with the Malvern laser particle size analyzer software (v7.12). Additionally, the elemental valence distributions were recorded using an x-ray photoelectron spectrometer (PHI 5000 VersaProbe III).

#### Ion release

To evaluate the release behavior of copper ions, CuPB nanozymes (1 mg) were dispersed in 2 ml of phosphate-buffered saline (PBS; pH 7.4). At predetermined time, 1 ml of the release medium was withdrawn for analysis and immediately replaced with an equal volume of fresh, prewarmed PBS to maintain sink conditions. The concentration of released Cu^2+^ was quantified using ICP-MS, (Agilent 7800, USA).

### Determination of the ROS-scavenging capacity

#### DPPH-radical-scavenging assay

A 1-mg quantity of DPPH was dissolved in 25 ml of ethanol and then diluted to achieve an absorbance within the range of 0.6 to 1.0. Subsequently, PB and XPB at corresponding concentrations were added to the DPPH-containing solution. The mixtures were incubated at 37 °C in the dark for 30 min. After filtration through a filter membrane, the absorbance of different solutions was measured at 517 nm using a microplate reader (EPOCH2NS, BioTek). The DPPH-radical-scavenging capacity was calculated using the following equation, where A0 represents the absorbance of the control sample and A1 represents the absorbance when the sample is present at any given time:

$$\text{Radical}-\text{scavenging rate}=\left[\left(A0-A1\right)/A0\right]\times 100\%$$ (1)

#### ABTS^+^·-radical-scavenging assay

An aqueous solution of 7 mM ABTS was mixed with an aqueous solution of 2.45 mM potassium persulfate in the dark for 16 h to obtain an ABTS^+^· radical aqueous solution. Then, PB and XPB nanozymes at corresponding concentrations were added to the ABTS-containing solution. The mixtures were incubated at 37 °C in the dark for 30 min. After filtration through a filter membrane, the absorbance of the mixtures was monitored at 734 nm using a microplate reader (EPOCH2NS, BioTek). The ABTS-radical-scavenging capacity was calculated using the following equation, where A0 represents the absorbance of the control sample and A1 represents the absorbance when the sample is present at any given time:

$$\begin{array}{c} \text{Radical}-\text{scavenging rate}=\left[\left(A0-A1\right)/A0\right]\times 100\% \end{array}$$ (2)

#### H_2_O_2_-scavenging assay

The H_2_O_2_-scavenging capacity of different amounts of PB and CuPB nanozymes was detected using an H_2_O_2_ content detection kit (Solarbio, China).

#### ·O_2_^−^-scavenging assay

The ·O_2_^−^-scavenging capacity of different amounts of PB and CuPB nanozymes was detected using an SOD enzyme activity kit (Beyotime, China). Additionally, the removal of ·O_2_^−^ by the nanozymes was detected using electron spin resonance spectroscopy (Bruker EMXplus, Germany). First, ·O_2_^−^ was generated using a TiO_2_/ultraviolet (UV) system under xenon lamp UV light as the control group. Then, PB or CuPB was added to the abovementioned mixture and incubated for 30 min. The residual ·O_2_^−^ was trapped using 5,5-dimethyl-1-pyrroline-*N*-oxide (DMPO).

#### ·OH-scavenging assay

The ·OH-scavenging capacity of different amounts of PB and CuPB nanozymes was detected using an ·OH content detection kit (Solarbio, China). Moreover, the removal of ·OH by the nanozymes was detected using electron spin resonance spectroscopy (Bruker EMXplus, Germany). First, ·OH was generated using an H_2_O_2_/UV system under xenon lamp UV light as the control group. Then, PB or CuPB was added to the abovementioned mixture and incubated for 30 min. The residual ·OH was captured using DMPO.

### DFT calculations

First-principles calculations based on DFT were performed using the Vienna ab initio Simulation Package (VASP). The Perdew–Burke–Ernzerhof functional within generalized gradient approximation (GGA) was used to describe electron exchange and correlation effects. To address the limitations of standard GGA in treating strongly correlated systems, the GGA+U method was employed, with effective Hubbard U values of 3.0 eV for Fe(II) and 7.0 eV for Fe(III). The van der Waals interactions were accounted for using Grimme’s DFT-D3 correction. A vacuum layer of 15 Å was introduced along the *z*-direction to eliminate interactions between periodic images. The plane-wave energy cutoff was set to 450 eV. Brillouin zone sampling was performed using a 2 × 2 × 1 Monkhorst–Pack *k*-point mesh. Structural relaxations were carried out until the maximum force on each atom was below 0.04 eV/Å, and the electronic energy convergence threshold was set to 10^−6^ eV. The Gibbs free energy (Δ*G*) of each intermediate was calculated using the following expression:

$$∆G=∆E+∆E_{\mathrm{ZPE}}+∆H_{0\to T}-T∆S$$ (3)

where Δ*E* denotes the change in electronic energy obtained from DFT and Δ*E*_ZPE_, $∆H_{0\to T}$, and Δ*S* are the changes in zero-point energy, enthalpy, and entropy at standard conditions (*T* = 298 K, at standard potential vs normal hydrogen electrode).

### Cell experiments

#### Cell culture and CCK-8 assay

The HUVECs were purchased from the Shanghai Cell Bank of the Chinese Academy of Sciences. The rat cardiomyocyte cell line (H9c2) was obtained from Procell Life Science & Technology Co., Ltd. (China), and the mouse myoblast cell line (C2C12) was purchased from Beijing Zhongyuan Heju Biotechnology Co., Ltd., the authorized American Type Culture Collection distributor in China (CRL1772). The cells were cultured in high-glucose Dulbecco’s modified Eagle medium (DMEM) supplemented with 10% fetal bovine serum and 1% penicillin–streptomycin in a humidified incubator at 37 °C with 5% CO_2_.

H9c2 cells, HUVECs, and C2C12 cells were seeded into 96-well plates (5 × 10^3^ cells per well) and cultured for 12 h. Subsequently, the cells were incubated with fresh DMEM or fresh DMEM containing different concentrations of PB and CuPB (concentration gradients: 200, 100, 50, 25, 12.5, 6.25, 3.125, 1.5625, and 0.78125 μg/ml) for 24 h. Cell viability was then assessed using the CCK-8 assay kit (YEASEN, China). Briefly, after removing the culture medium, fresh medium containing 10 μl of the CCK-8 reagent per well was added, and the cells were incubated at 37 °C for 1.5 h. The absorbance was then measured at a wavelength of 450 nm using a microplate reader (EPOCH2NS, BioTek) to evaluate the cytotoxic effects of the materials.

To verify the ability of PB and CuPB nanozymes to repair cardiomyocyte damage, H9c2 cells were seeded into 96-well plates (1 × 10^4^ cells per well) and cultured for 12 h. A cardiomyocyte injury model was established by adding 1,000 μM H_2_O_2_ to the culture medium and incubating for 4 h. Subsequently, the cells were further incubated with fresh DMEM or fresh DMEM containing PB and CuPB nanozymes (3.125 μg/ml) for 4 h. Cell viability in different groups was then detected using the CCK-8 reagent.

#### Cellular uptake

A 200-μl aliquot of rhodamine B-labeled solution (5 mg/ml) was added to a reaction flask containing 1 ml of PB or CuPB aqueous solution (1 mg/ml). The mixture was stirred at room temperature overnight, followed by centrifugation to obtain rhodamine PB/CuPB for fluorescence analysis. HUVECs were seeded onto circular coverslips with a diameter of 8 mm and cultured for 12 h. Subsequently, the cells were incubated with rhodamine-labeled PB or CuPB nanozymes for 4 h. The cell nuclei were then stained with Hoechst (Beyotime, China), and the samples were observed using a fluorescence microscope (Axio Vert.A1, Germany).

For lysosome colocalization observation, HUVECs (2 × 10^5^ cells per well) were seeded into culture dishes and incubated at 37 °C for 24 h. After the cells adhered to the dish surface, the culture medium was replaced with fresh medium containing rhodamine-labeled PB or CuPB nanozymes, and the cells were incubated for 4 h. After washing with PBS 3 times, the endosomes/lysosomes were stained with LysoTracker Green for 40 min, and the cell nuclei were stained with Hoechst for 20 min. The localization of the materials and lysosomes was observed and recorded using a confocal laser scanning microscopy (ZEISS LSM 900, Germany).

#### DCFH-DA staining

H9c2, HUVECs, and C2C12 were seeded into 48-well plates at a density of 2 × 10^4^ cells per well and cultured for 12 h. A 1,000 μM concentration of H_2_O_2_ was added to the culture medium, and the cells were incubated for 4 h. Then, the cells were further incubated with fresh DMEM or DMEM containing PB and CuPB nanozymes for 4 h. Subsequently, DCFH-DA (YEASEN, China) staining was used to assess the intracellular ROS levels. Briefly, the DCFH-DA fluorescent probe was added to the culture medium, and the cells were incubated at 37 °C for 30 min. Then, the cells were washed twice with DMEM and stained with Hoechst (Beyotime, China) for 5 min. The fluorescence signals were detected using a fluorescence microscope (Axio Vert.A1, Germany) and analyzed with the ImageJ software (National Institutes of Health, USA).

#### TUNEL staining

H9c2 and C2C12 were seeded into 48-well plates at a density of 1 × 10^4^ cells per well and cultured for 12 h. A 1,000 μM concentration of H_2_O_2_ was added to the culture medium, and the cells were incubated for 4 h. Then, the cells were further incubated with fresh DMEM or DMEM containing PB and CuPB for 4 h. Apoptotic cells were detected using a TUNEL staining kit (Beyotime, China) according to the manufacturer’s instructions. Briefly, the treated H9c2 cells were fixed in an immunostaining fixation solution for 30 min and then washed with PBS. Subsequently, the cells were incubated with an enhanced immunostaining permeabilization buffer for 5 min and then with the TUNEL test solution at 37 °C for 1 h. Finally, the cells were counterstained with 4′,6-diamidino-2-phenylindole (DAPI, Solarbio, China), and TUNEL-positive cells were observed using a fluorescence microscope (Axio Vert.A1, Germany). The total cell nuclei (stained blue with DAPI) and TUNEL-positive cell nuclei (stained green) in 5 randomly selected fields of view in each H9c2 sample were counted, and the apoptosis index was calculated as (number of TUNEL-positive cell nuclei/total number of cell nuclei) × 100%.

#### Tube-formation assay

A Matrigel matrix (ABW Matrigel matrix, China) was placed on an ice and completely thawed at 4 °C. Then, the thawed Matrigel was added to the bottom of pre-cooled 48-well plates (150 μl per well) and incubated at 37 °C for 30 min. After that, a suspension containing HUVECs and PB or CuPB nanozymes was seeded onto the Matrigel-embedded 48-well plates at a density of 4 × 10^4^ cells per well. The cells were incubated at 37 °C for 4 h and photographed using a microscope. The ability to form a vascular network was quantified using the ImageJ software (National Institutes of Health, USA).

#### Immunofluorescence staining of HIF-1α

HUVECs were seeded into culture dishes at a density of 2 × 10^5^ cells per well and incubated for 12 h to allow for cell adhesion and initial adaptation to the culture environment. Subsequently, H_2_O_2_ was added to the culture medium at a final concentration of 1,000 μM, and the cells were incubated for 4 h to induce a specific cellular stress response. After this incubation period, the medium was replaced with either fresh DMEM or DMEM supplemented with PB and CuPB nanozymes, and the cells were further incubated for an additional 4 h. Following these treatment steps, fluorescence staining for HIF-1α was performed.

#### Quantitative real-time polymerase chain reaction

H9c2, C2C12, and HUVECs were seeded into 12-well plates at a density of 1.5 × 10^5^ cells per well, respectively. Subsequently, H_2_O_2_ at a concentration of 1,000 μM was added to the culture medium, and the cells were incubated for 4 h. After that, the cells were further incubated for 4 h with either fresh DMEM or DMEM containing PB or CuPB nanozymes. In addition, HUVECs were seeded into 12-well plates at a density of 1.5 × 10^4^ cells per well and then incubated with either fresh DMEM or DMEM containing PB or CuPB nanozymes for 72 h. RNA was extracted from each of the 3 cell types after collection. Quantitative analysis of all complementary DNA templates was performed using a fluorescent real-time PCR system, according to the manufacturer’s instructions (ChamQ SYBR qPCR Master Mix, Q311).

#### Transcriptome sequencing analysis

RNA sequencing (provided by LC-Bio Technology Co., Ltd., Hangzhou, China) was employed to identify DEGs. Subsequently, the biological functions and signaling pathways associated with these DEGs were investigated through Gene Ontology enrichment analysis and KEGG pathway enrichment analysis. Additionally, pathway enrichment maps were generated to visually represent the results.

### In vivo therapeutic performance

#### Construction of mouse HI and nanozyme administration

Male Institute of Cancer Research (ICR) mice (10 weeks old) intended for the establishment of a CLI model were obtained from the Zhejiang Provincial Laboratory Animal Center. The experimental procedures were approved by the Animal Research and Ethics Committee of the Wenzhou Institute, University of Chinese Academy of Sciences (Approval No.: WIUCAS24110701). Initially, the mice were anesthetized with 2% isoflurane. Subsequently, an incision was made in the skin at the inguinal region of the left hind limb to expose the left femoral artery. The proximal and distal ends of the artery were ligated using 8-0 surgical suture. The segment of the femoral artery between the 2 ligation points was then excised, and the vessel was completely transected between the 2 sutures. The contralateral hind limb served as an internal control. The muscle and skin were subsequently sutured continuously using 5-0 surgical sutures.

After surgery, the mice were allowed to recover on a warm pad and were housed in cages with free access to food and water. The control group underwent the same procedures, except for the ligation of the femoral artery; all other steps were consistent with those of the HI model, and this group was designated as the sham group. For the experimental groups, PB or CuPB nanozymes (10 mg/kg based on mouse body weight) were intramuscularly injected into the periwound area (4-site injection, 10 μl per site) immediately after surgery using a widely used commercial human serum albumin hydrogel. These groups were defined as the PB_i.m._ and CuPB_i.m._ groups, respectively. Simultaneously, CuPB nanozymes (10 mg/kg based on mouse body weight) dispersed in PBS were tail vein injected, designated as the CuPB_i.v._ group. The control group without any treatment was designated as the NT group.

#### Blood flow imaging

Blood flow in the mouse hind limbs was measured on days 0, 7, 14, 21, and 28 after surgery using a laser Doppler perfusion imager (Lumina 3, Revvity, USA). The obtained images were analyzed, and the perfusion ratio was calculated as the blood flow in the left hind limb (ligated) divided by that in the right hind limb (unligated) to evaluate the blood supply in the ischemic and healthy hind limbs. After anesthesia, the mice were placed on a 37 °C warm plate to minimize variations in blood flow caused by ambient temperature.

#### Biodistribution and metabolism

To monitor the degradation of the nanozymes injected in situ into the muscle, Cy5.5-labeled PB and CuPB nanozymes were prepared and injected into the muscle of mice after model construction. At set time points, the mice were anesthetized with isoflurane, and the fluorescent signal in the legs was recorded using an in vivo imaging system (IVIS Lumina III, PerkinElmer, USA). Subsequently, the mice were euthanized, and tissues such as the gastrocnemius muscle, heart, liver, spleen, lungs, and kidneys were harvested for ex vivo imaging. Similarly, to monitor the in vivo metabolism of CuPB nanozymes after tail-vein injection, Cy5.5-labeled CuPB nanozymes were administered to the model mice by tail-vein injection. The in vivo fluorescence distribution in the mice was recorded at predetermined time points using the IVIS imaging system. Finally, the mice were euthanized, and tissues including the gastrocnemius muscle, heart, liver, spleen, lungs, and kidney were collected for ex vivo imaging.

#### Construction of mouse MI and nanozyme administration

Male ICR mice (8 weeks old) intended for MI model construction were procured from the Zhejiang Provincial Laboratory Animal Center. The experimental procedures were approved by the Animal Research and Ethics Committee of the Wenzhou Institute, University of Chinese Academy of Sciences (Approval No.: WIUCAS23041202). For the MI model, mice were anesthetized with sodium pentobarbital (50 mg/kg) and ventilated using a rodent ventilator (GT-43006-4015-T3, VentElite, Germany) at a respiratory rate of 110 breaths per minute and a tidal volume of 100 μl. Subsequently, the mice were repositioned from a supine to the right lateral position. The heart was fully exposed through a left minithoracotomy. An 8-0 surgical suture was passed beneath the LAD, located 2 to 3 mm distal to the junction of the left auricle and the conus arteriosus. The ribs, muscles, and skin were then continuously sutured using a 5-0 surgical suture. After surgery, the mice were allowed to recover on a warm pad and were housed in cages with free access to moist food and water. The control group underwent the same procedures, except for the ligation of the LAD; all other steps were consistent with those of the MI model, and this group was designated as the sham group [60]. For the experimental groups, PB or CuPB nanozymes (10 mg/kg based on mouse body weight) were immediately intramyocardially injected into the peri-infarct region (2 injection sites, 10 μl per site) after surgery using a widely used commercial human serum albumin hydrogel, defined as the PB_i.m._ and CuPB_i.m._ groups. Mice in the CuPB_i.v._ group received a tail-vein injection of CuPB nanozymes (10 mg/kg based on mouse body weight) dispersed in PBS. The group that underwent only MI surgery without any treatment was defined as the NT group.

#### Echocardiographic assessment

Cardiac function was monitored via echocardiography on day 28 post-MI using a high-resolution small-animal ultrasound imaging system (Fujifilm VisualSonics, Canada). Briefly, mice were anesthetized under 1.5% isoflurane delivered via an anesthesia machine, and their chest hair was removed with depilatory cream. M-mode echocardiography was performed using the Vevo 3100 imaging system equipped with an MX400, 30-MHz transducer. After obtaining a short-axis view, cardiac parameters, including the percentages of LVEF and LVFS, were measured using the Vevo LAB software (version 3.1.1).

#### Histological analysis

On day 7 or 28 following MI or on day 28 following HI, the mice were euthanized, and their hearts and gastrocnemius muscles were harvested. Initially, the weights of the gastrocnemius muscle samples were measured. Subsequently, the samples were fixed overnight in 4% paraformaldehyde and then embedded in paraffin. The heart samples were sectioned into 5-μm-thick consecutive paraffin slices along a direction perpendicular to the apex and passing through the infarcted area, while the gastrocnemius muscle samples were sectioned transversely. After deparaffinization and rehydration, the sections were subjected to Masson’s trichrome staining, H&E staining, CD31 immunofluorescent staining, TUNEL staining, and cleaved caspase-3 immunohistochemical staining as needed.

### Statistical analysis

All results are presented as mean ± standard error of the mean. For comparisons involving more than 2 independent samples, one-way analysis of variance (ANOVA) with Tukey’s post hoc test was conducted to analyze the data using the GraphPad Prism 9 software (La Jolla, CA, USA), under the assumption of equal variances among groups. In cases where the variances were unequal, Welch’s ANOVA was employed instead. Statistical significance was defined as *P* < 0.05, with significance levels denoted as *P < 0.05, **P < 0.01, and ***P < 0.001.

## Ethical Approval

The experimental procedures were approved by the Animal Research and Ethics Committee of the Wenzhou Institute, University of Chinese Academy of Sciences (Approval No.: WIUCAS23041202).

## Data Availability

All data are available in the main text or the Supplementary Materials.
