# Supplementary material for: Doping-Engineered Proangiogenic Nanozymes Orchestrate Ischemic Tissue Regeneration via Cytoprotection and Revascularization
Source: Research (Wash D C). 2026 Apr 28;9:1260. doi: 10.34133/research.1260 (PMC13121891; doi:10.34133/research.1260)

Supplementary Materials for

Doping-Engineered Pro-Angiogenic Nanozymes Orchestrate Ischemic Tissue Regeneration via Cytoprotection and Revascularization

This PDF file includes:

Figure S1 to S30

Table S1


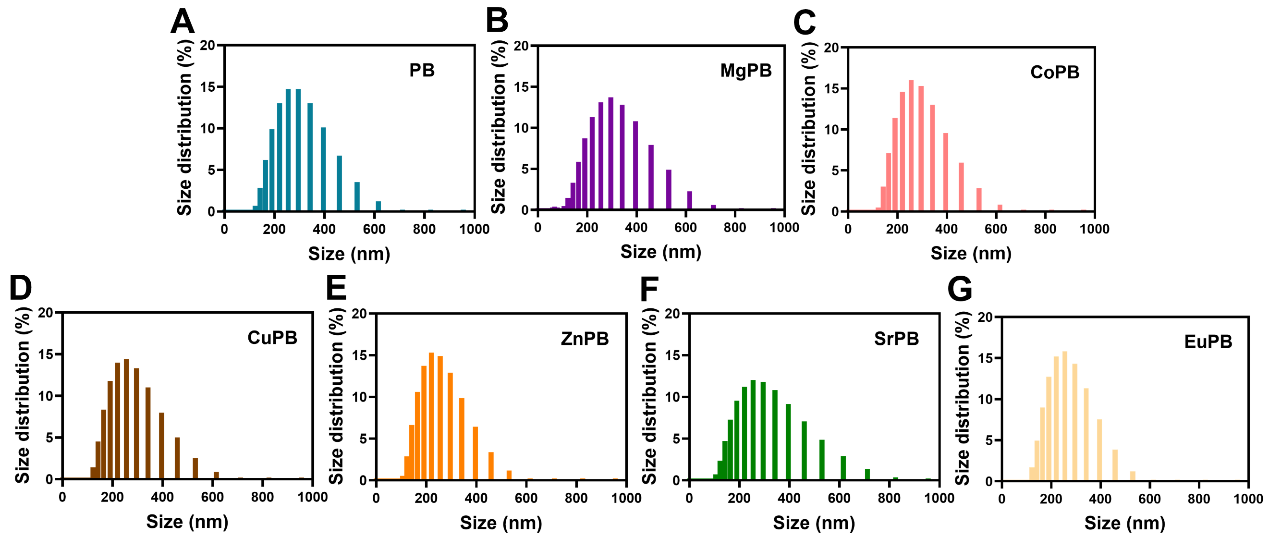


**Figure S1.** Hydrodynamic size distribution of XPB nanozymes determined by DLS.


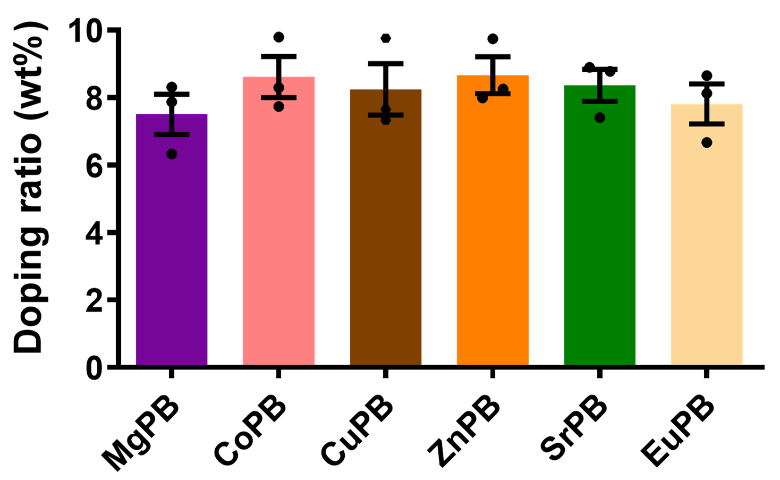


**Figure S2.** Doping ratio of X element to Fe in XPB nanozymes detected by ICP-MS. n = 3.


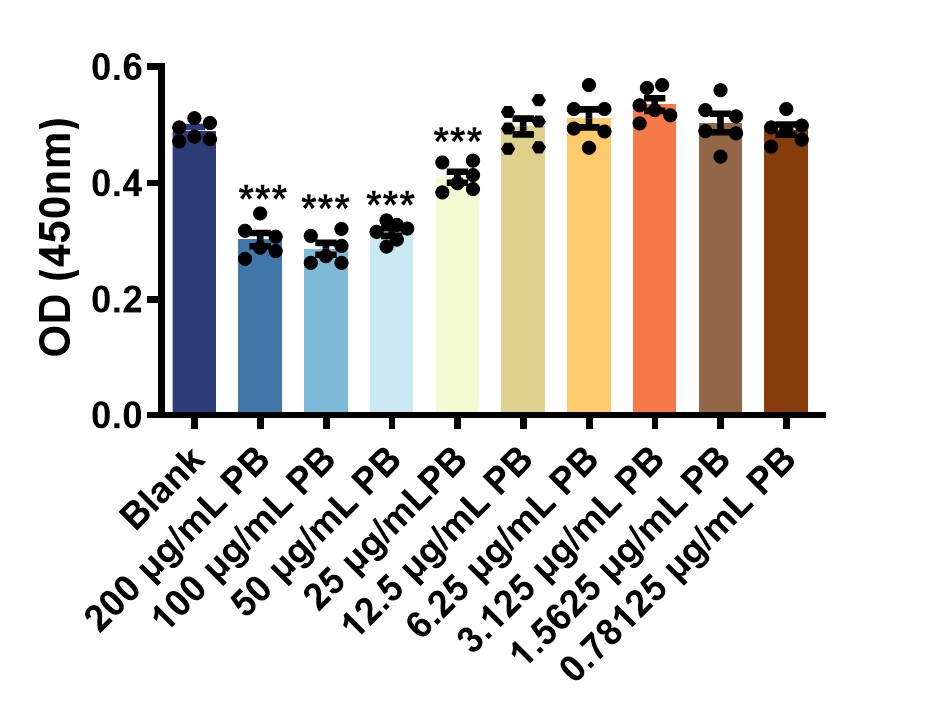


**Figure S3.** Cell viability of HUVECs exposed to varying concentrations of PB nanozymes, evaluated by CCK-8 assay. n = 6. * indicates significant difference compared to the PB group. ***P < 0.001.


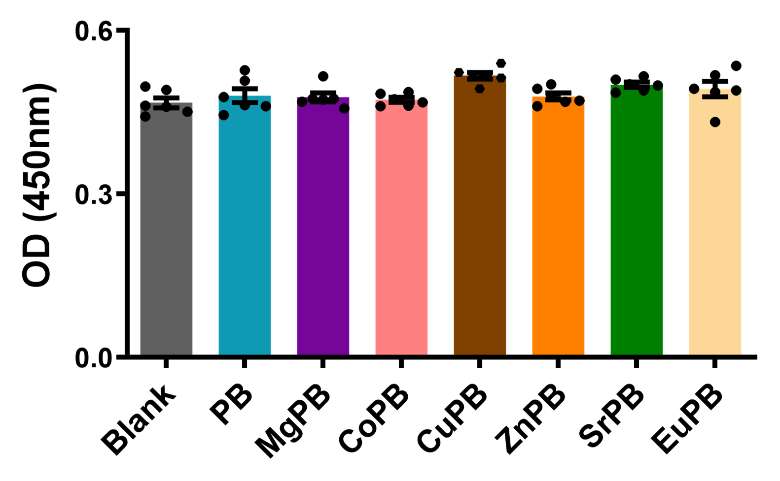


**Figure S4.** Cell viability of HUVECs treated with different XPB nanozymes at 3.125 μg/mL, assessed by CCK-8 assay. n = 6.


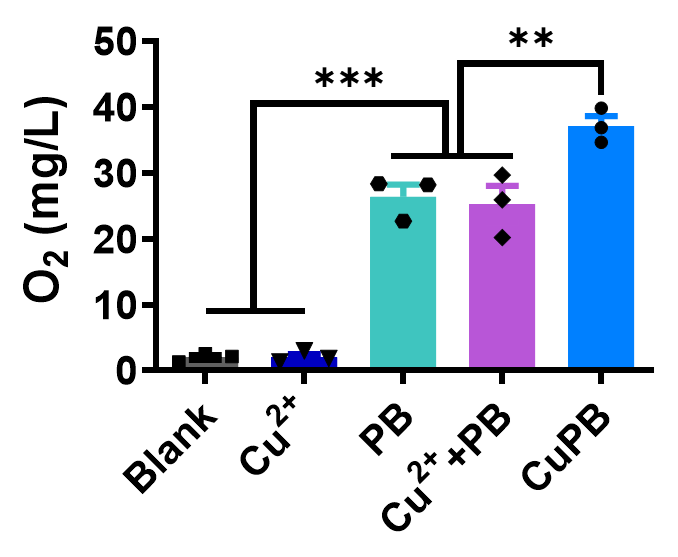


**Figure S5.** Comparison of catalase-like activity between CuPB and physical mixtures via dissolved oxygen monitoring. n = 3.


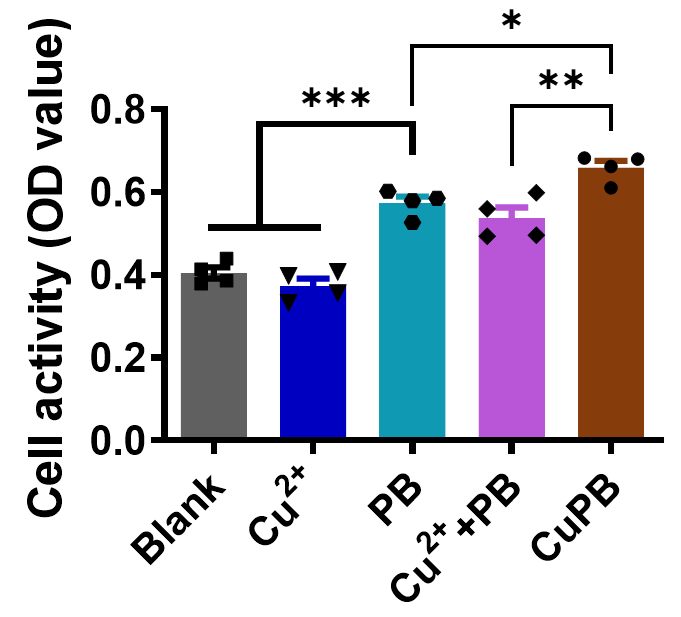


**Figure S6.** Cell protection efficacy of CuPB nanozymes compared to PB and Cu ion under H_2_O_2_-induced oxidative stress. n = 4.


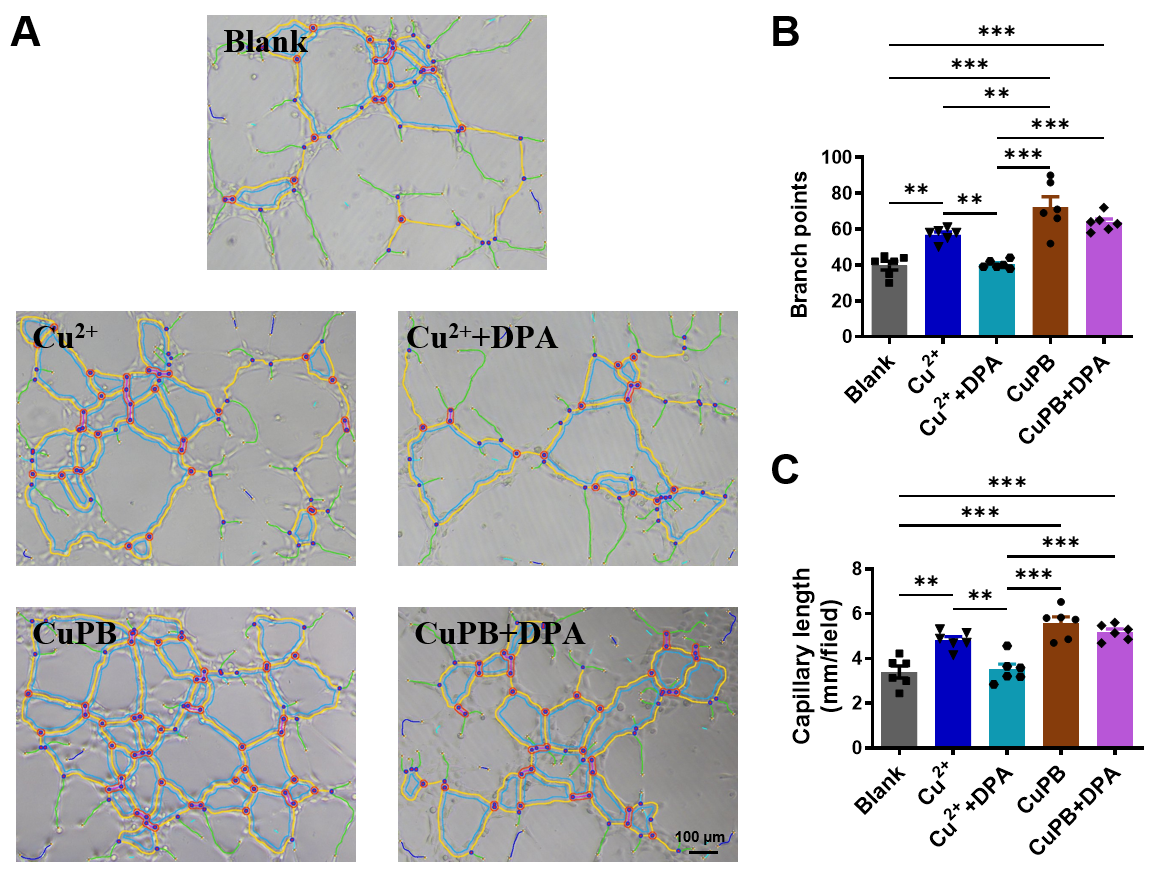


**Figure S7.** Validation of the copper ion-mediated angiogenic mechanism via D-Penicillamine (DPA) chelation. n = 6.


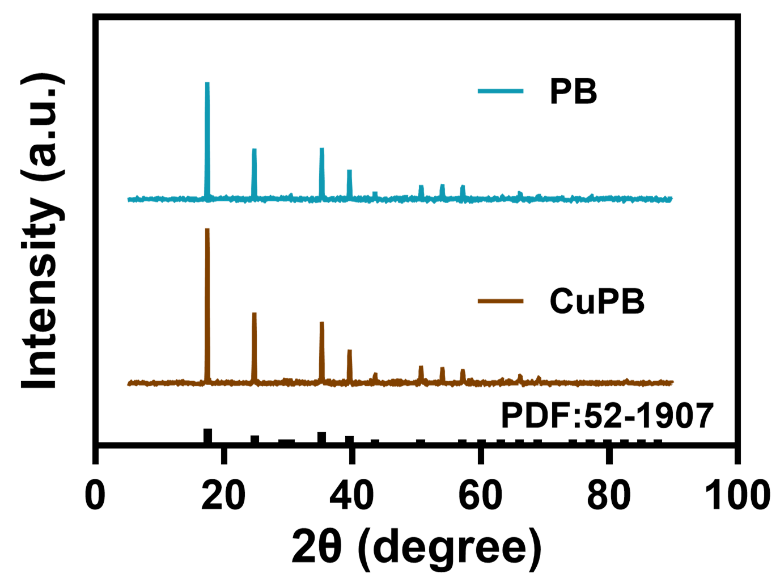


**Figure S8.** XRD patterns of PB and CuPB nanozymes.


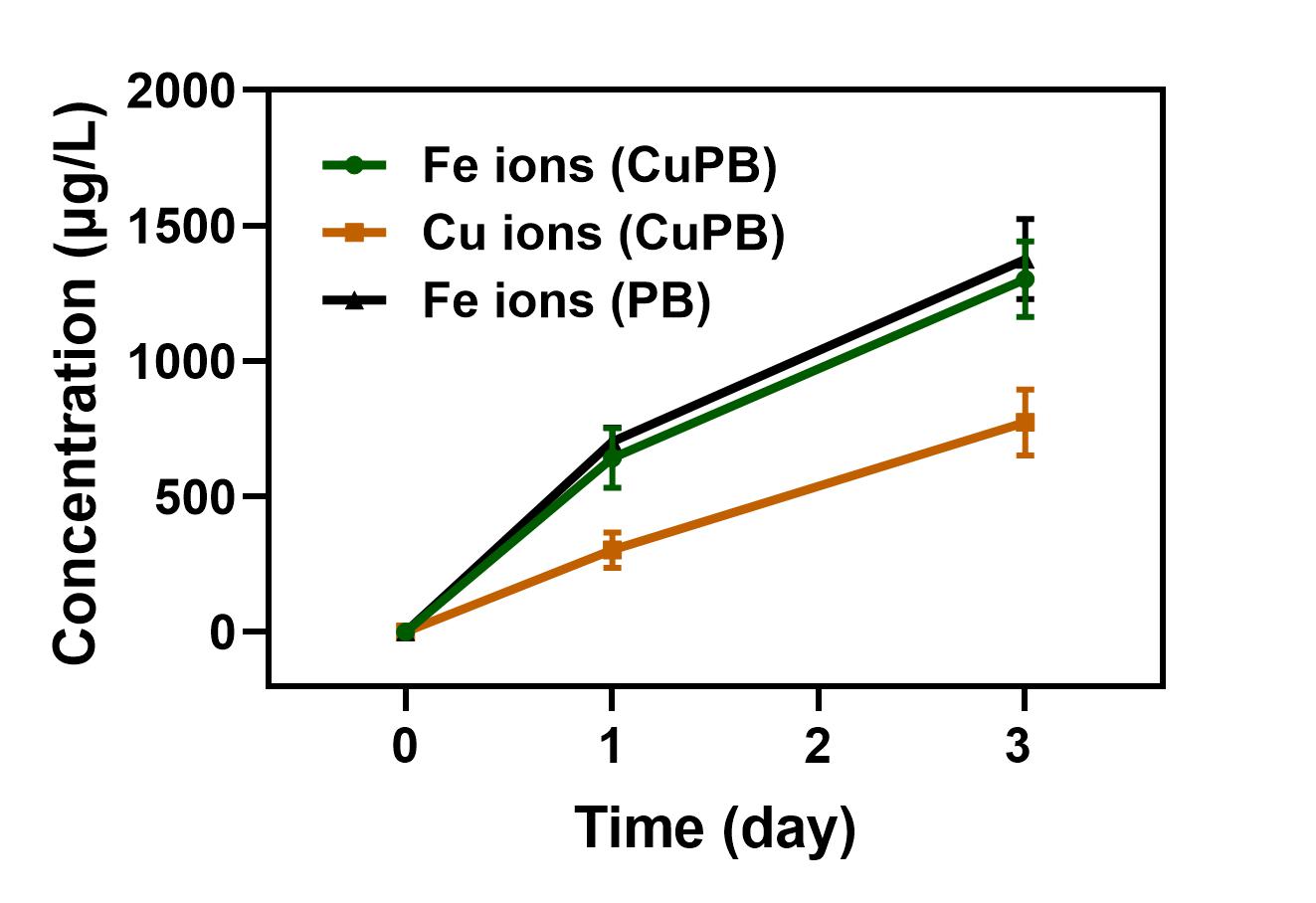


**Figure S9.** Ion release performance of CuPB and PB within 3 Days. n = 3.


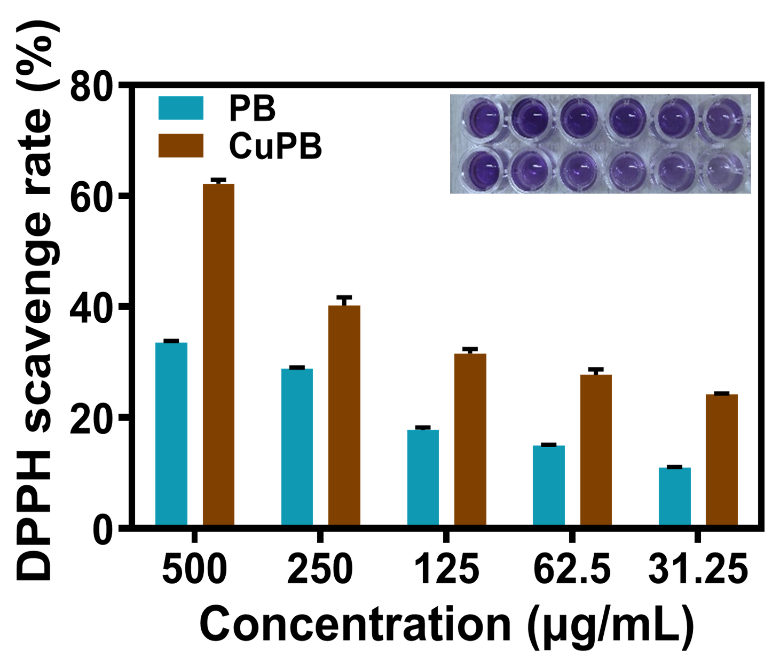


**Figure S10.** DPPH radical scavenging activity of PB and CuPB nanozymes at varying concentrations. n = 3.


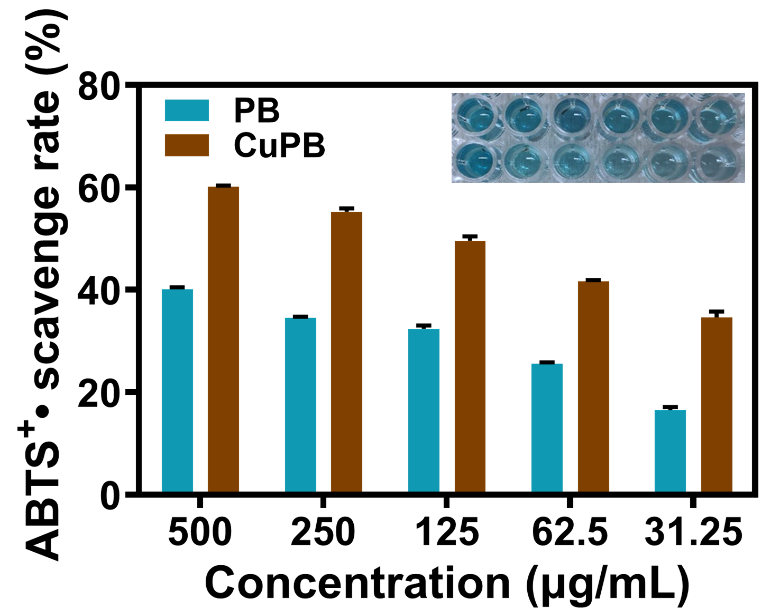


**Figure S11.** ABTS⁺• radical scavenging activity of PB and CuPB nanozymes at varying concentrations. n = 3.


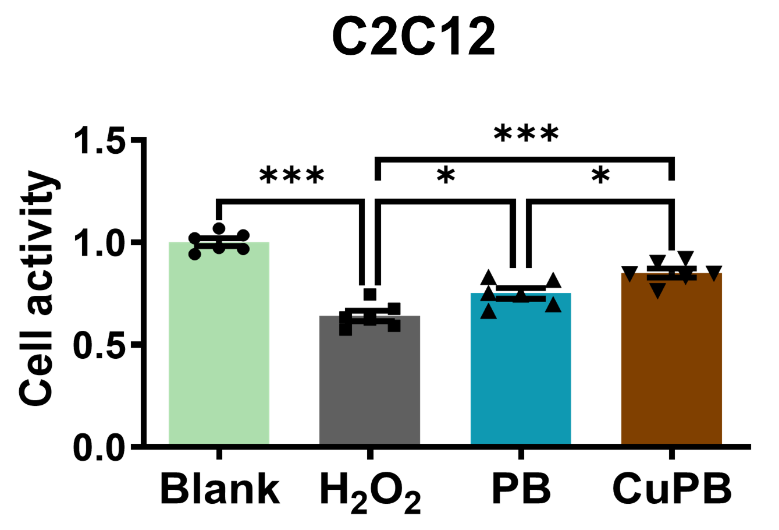


**Figure S12.** Cell viability of H_2_O_2_-injured C2C12 cells after treatment with PB or CuPB nanozymes, assessed by CCK-8 assay. n = 6.


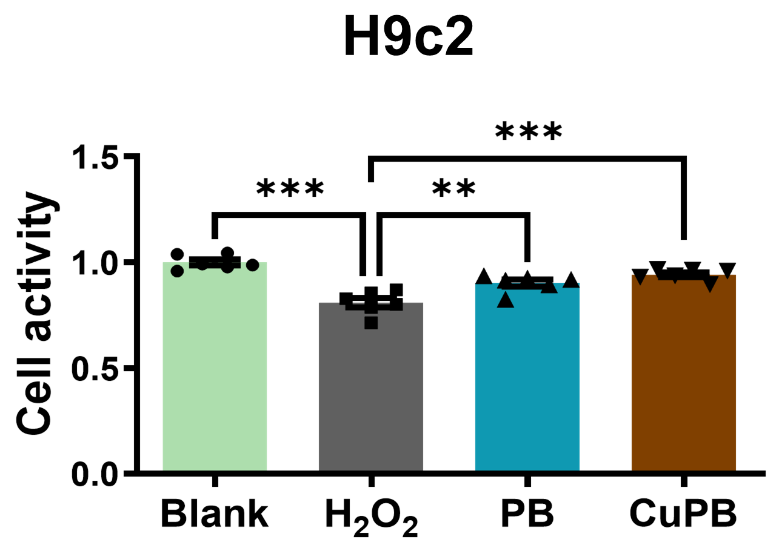


**Figure S13.** Cell viability of H_2_O_2_-injured H9c2 cardiomyocytes after treatment with PB or CuPB nanozymes, assessed by CCK-8 assay. n = 6.


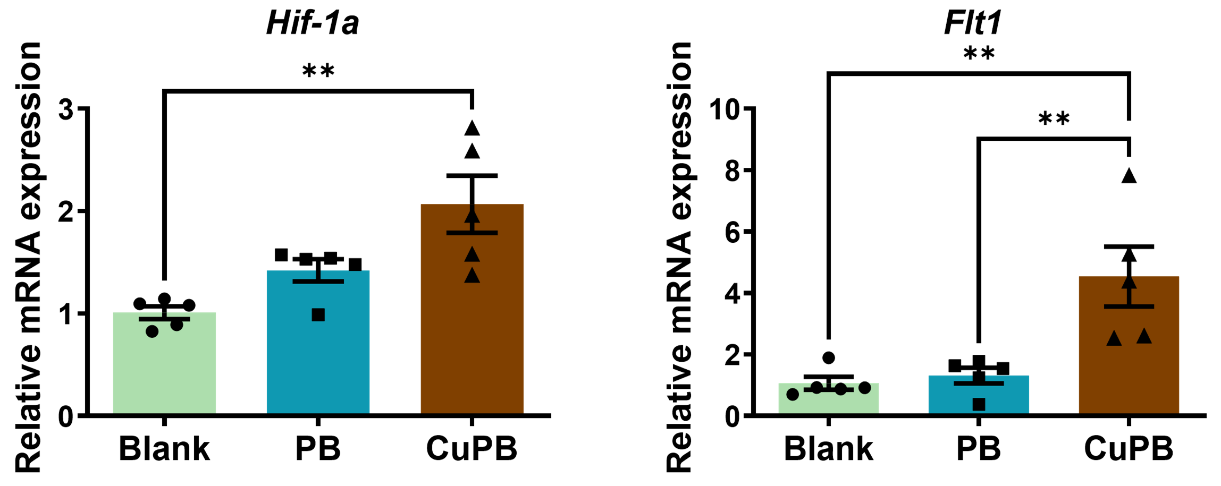


**Figure S14.** qRT-PCR analysis of angiogenesis-related genes (*Hif-1α* and *Flt1*) in HUVECs treated with PB or CuPB nanozymes. n = 5.


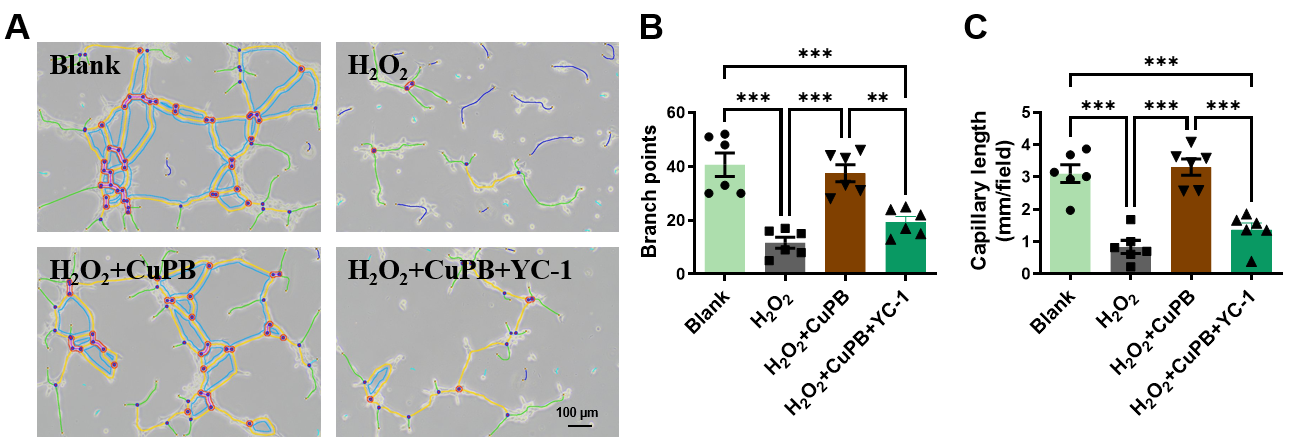


**Figure S15.** Verification of the HIF-1α-dependent angiogenic mechanism of CuPB nanozymes by Lificiguat (YC-1). n = 6.


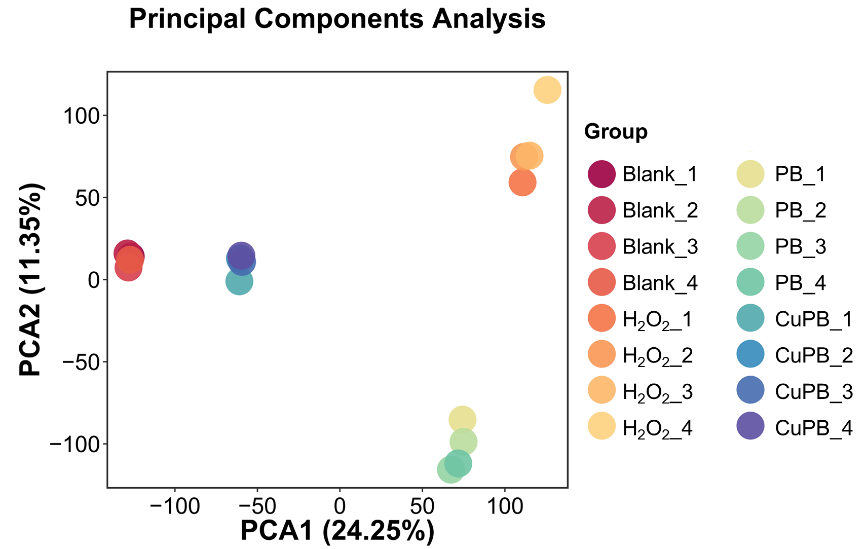


**Figure S16.** Principal component analysis (PCA) of transcriptomic profiles in Blank, H_2_O_2_, PB, and CuPB groups. Transcriptome sequencing analysis. n = 4.


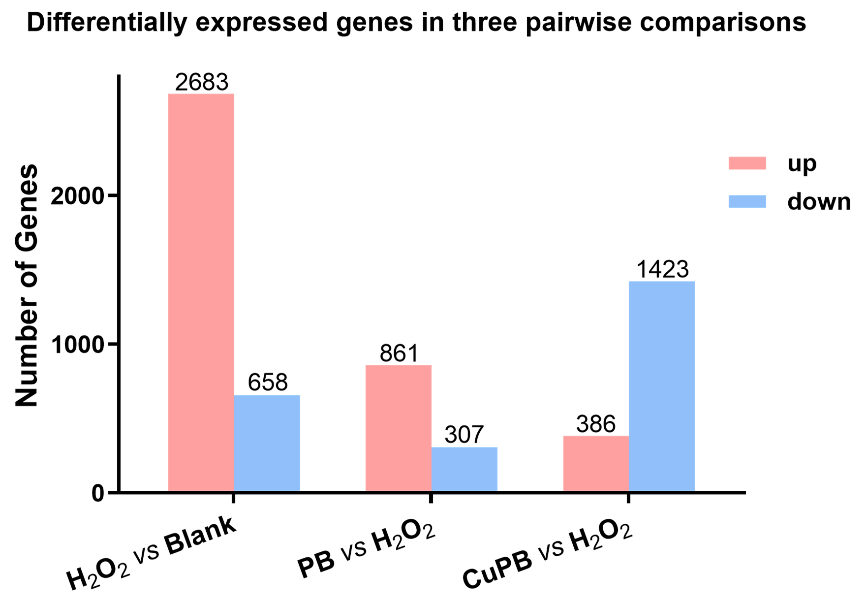


**Figure S17.** Number of differentially expressed genes in three pairwise comparisons (H_2_O_2_ *vs* Blank, PB *vs* H_2_O_2_, and CuPB *vs* H_2_O_2_). Transcriptome sequencing analysis. n = 4.


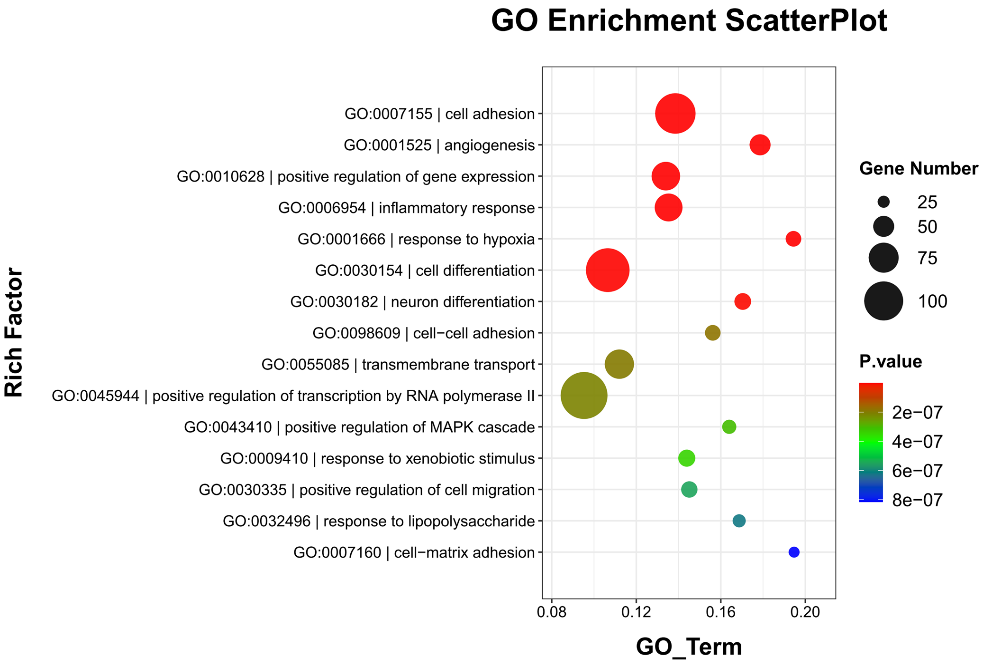


**Figure S18.** GO enrichment of biological processes in differentially expressed genes after H_2_O_2_-induced injury (H_2_O_2_ vs Blank). Transcriptome sequencing analysis. n = 4.


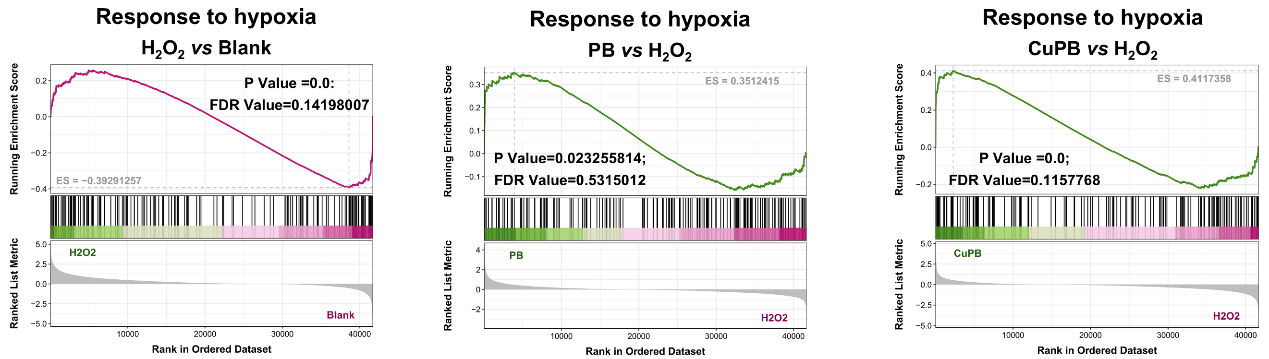


**Figure S19.** Comparison of the GO term “response to hypoxia” among three pairwise comparisons (H_2_O_2_ *vs* Blank, PB *vs* H_2_O_2_, CuPB *vs* H_2_O_2_). n = 4.


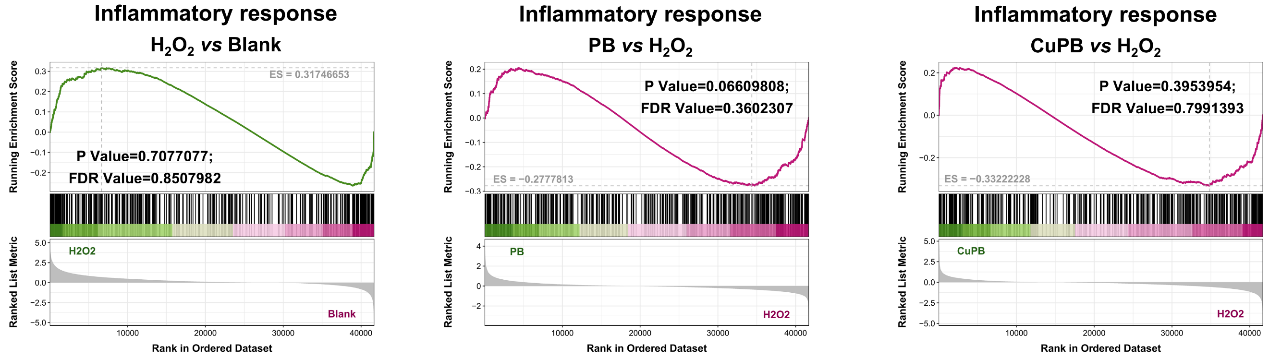


**Figure S20.** Comparison of the GO term “inflammatory response” among three pairwise comparisons (H_2_O_2_ *vs* Blank, PB *vs* H_2_O_2_, CuPB *vs* H_2_O_2_). Transcriptome sequencing analysis. n = 4.


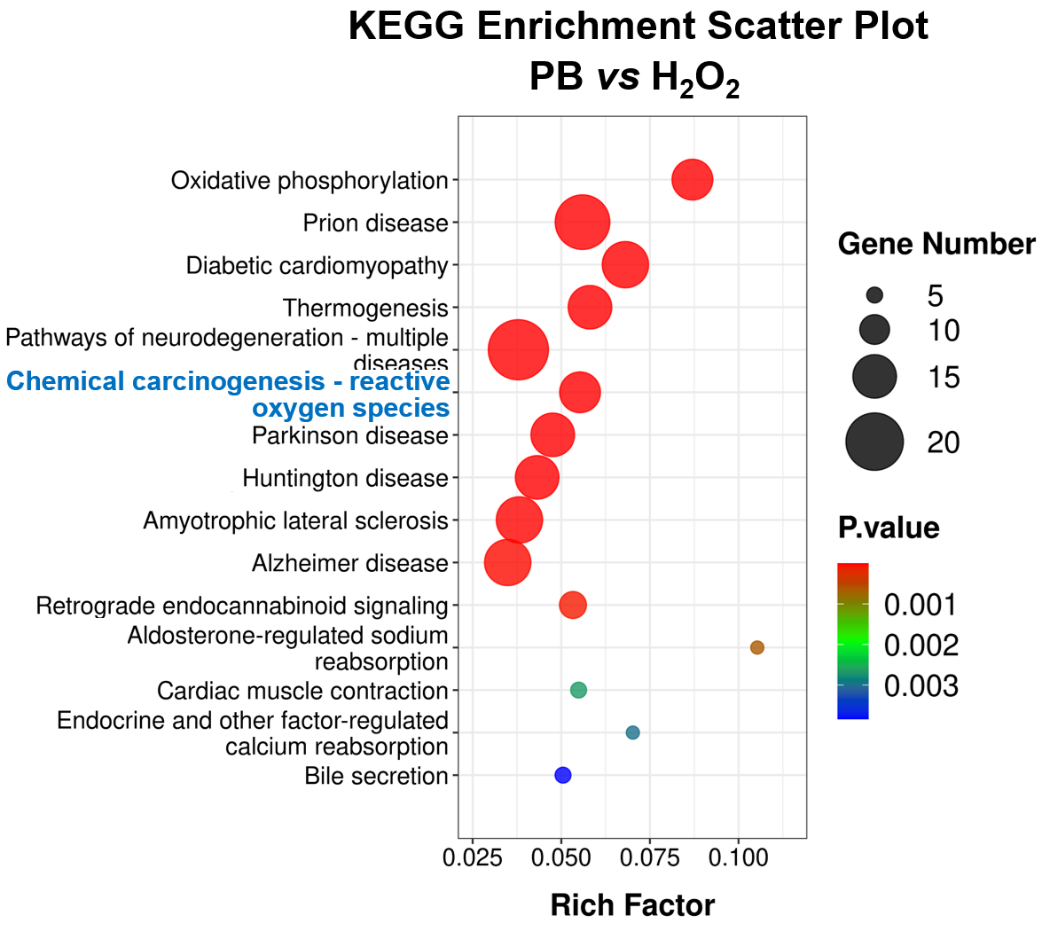


**Figure S21.** Top 15 KEGG signaling pathways enriched in differentially expressed genes between the PB treatment group and the H_2_O_2_ treatment group (PB vs H_2_O_2_). Transcriptome sequencing analysis. n = 4.


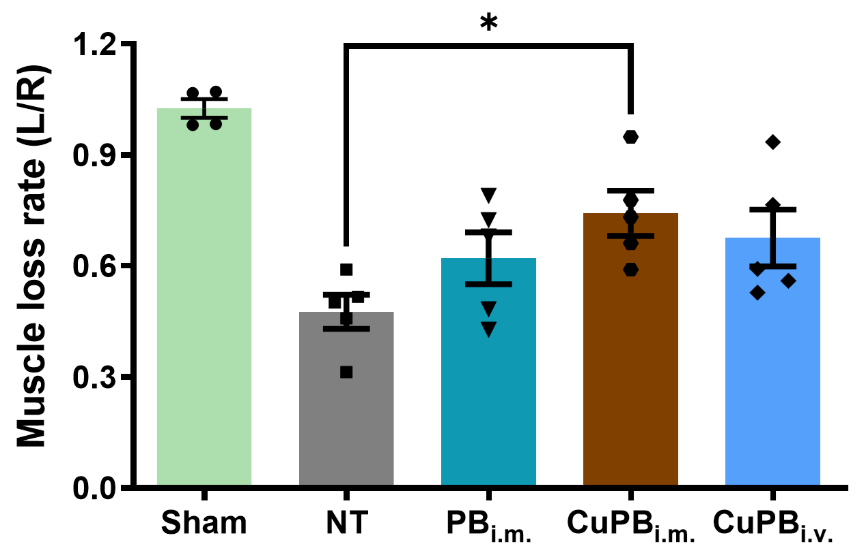


**Figure S22.** Quantitative analysis of gastrocnemius muscle loss ratio after different nanozyme treatments. n = 5.


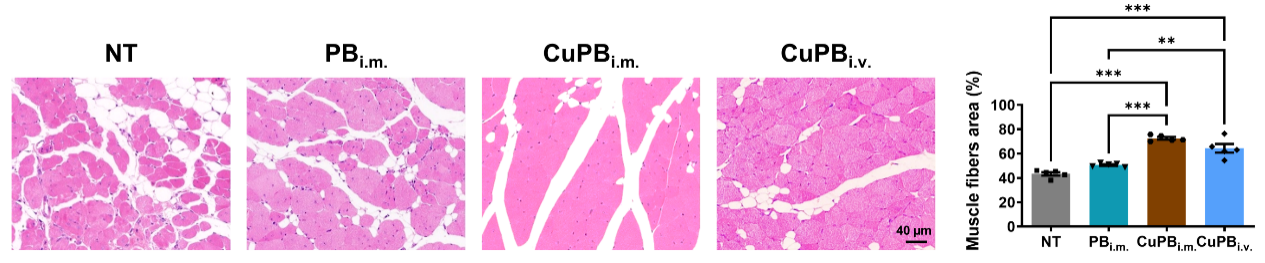


**Figure S23.** Representative H&E staining images and quantification of gastrocnemius fiber area after different nanozyme treatments. n = 5.


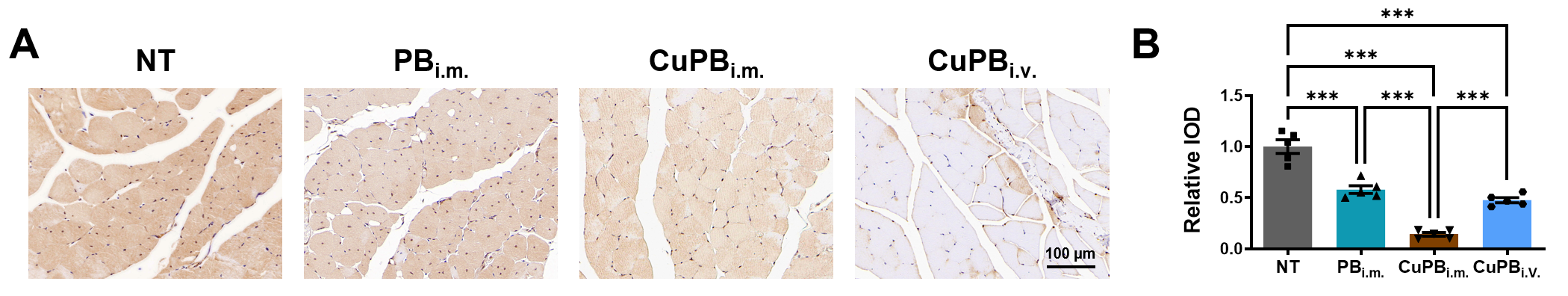


**Figure S24.** In vivo attenuation of oxidative stress-induced apoptosis in ischemic gastrocnemius muscle. (A) Representative Immunohistochemistry images of Cleaved Caspase-3 in gastrocnemius muscle sections. (B) Quantitative analysis of the Cleaved Caspase-3 positive cells. n = 5.


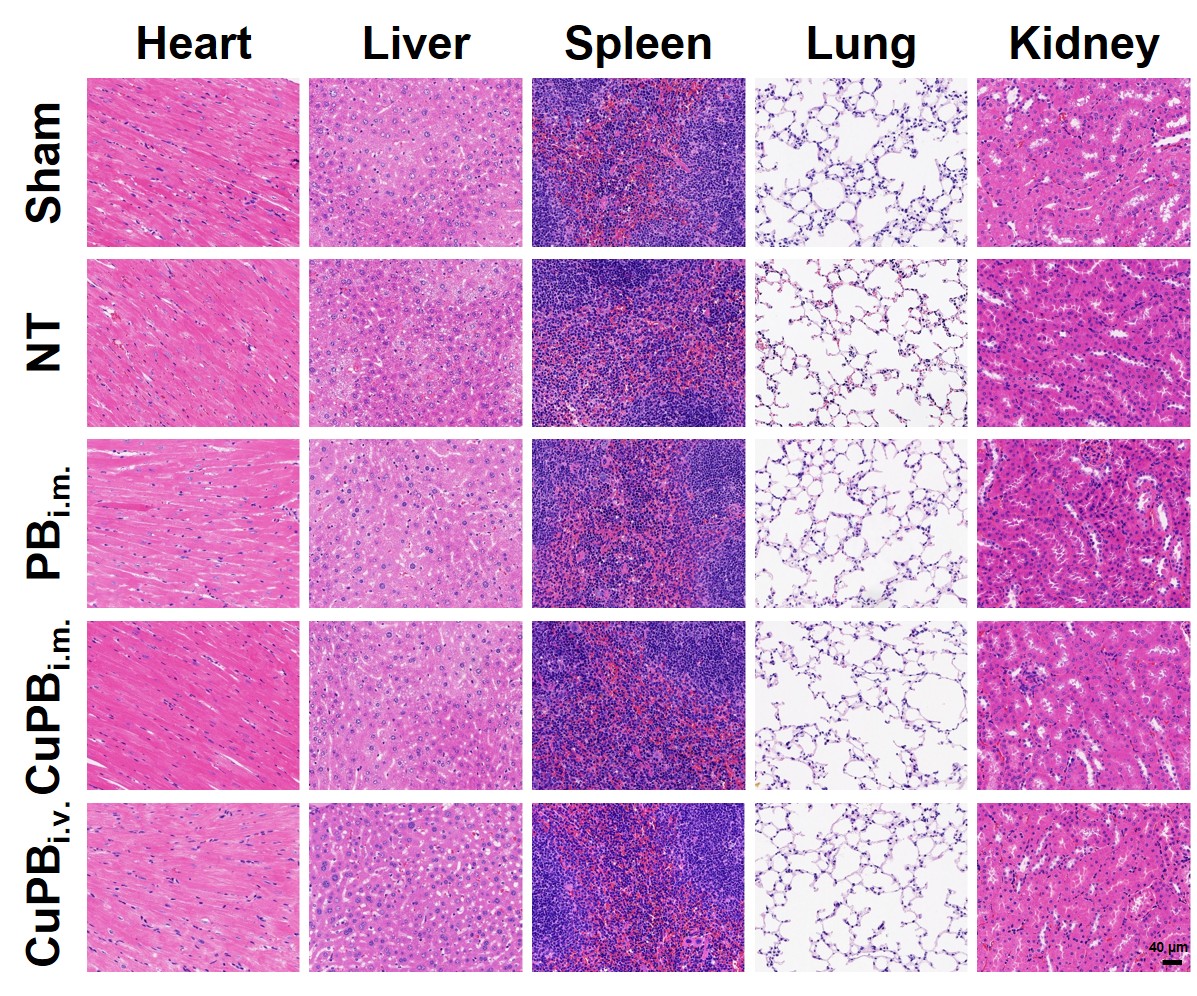


**Figure S25.** *In vivo* safety assessment of nanozyme treatment in mice with hindlimb ischemia.


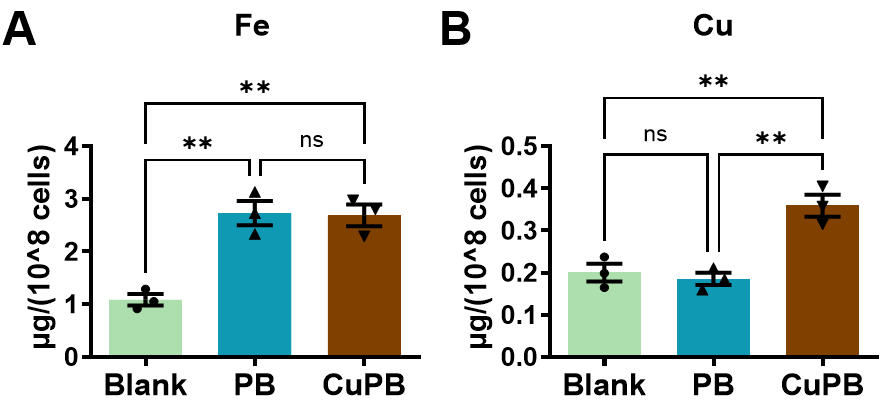


**Figure S26.** Quantitative assessment of cellular internalization efficiency via ICP-MS analysis.


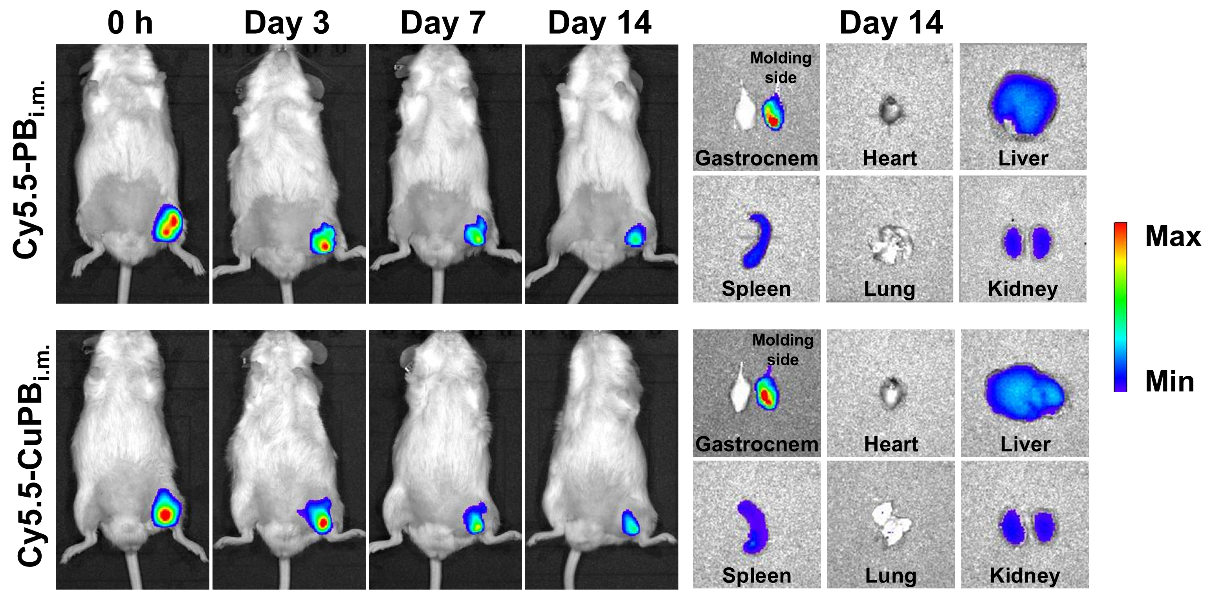


**Figure S27.** *In vivo* biodistribution and clearance after local injection of Cy5.5-labeled PB and CuPB nanozymes.


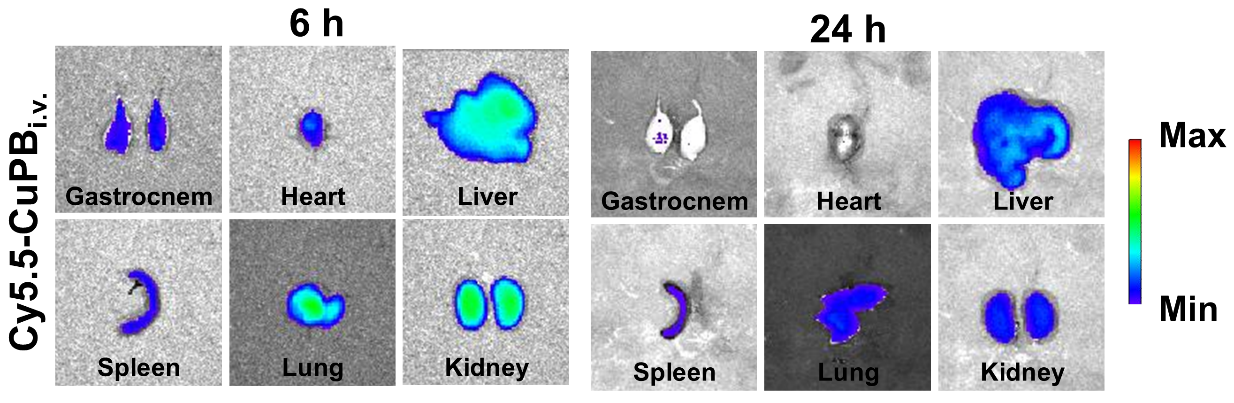


**Figure S28.** *In vivo* biodistribution and clearance after tail-vein injection of Cy5.5-labeled CuPB nanozymes.


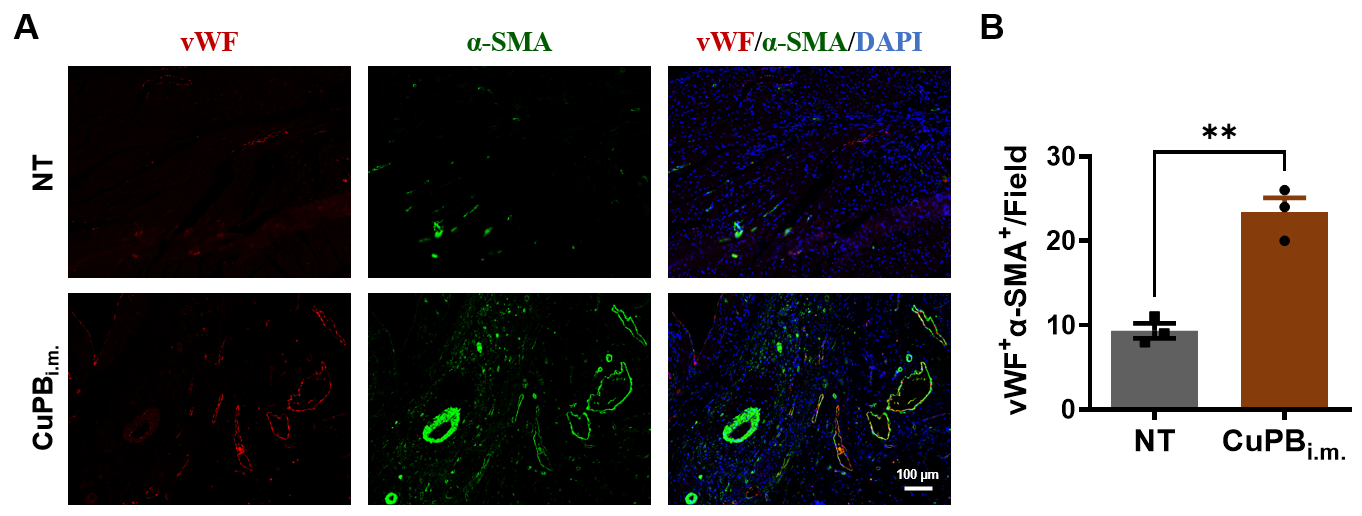


**Figure S29.** (A) Representative immunofluorescence images of the infarct border zone at day 28 post-MI, co-stained for vWF (endothelial marker, green) and α-SMA (smooth muscle/pericyte marker, red). (B) Quantitative analysis of vWF and α-SMA positive vessel density in the peri-infarct region. n = 3.


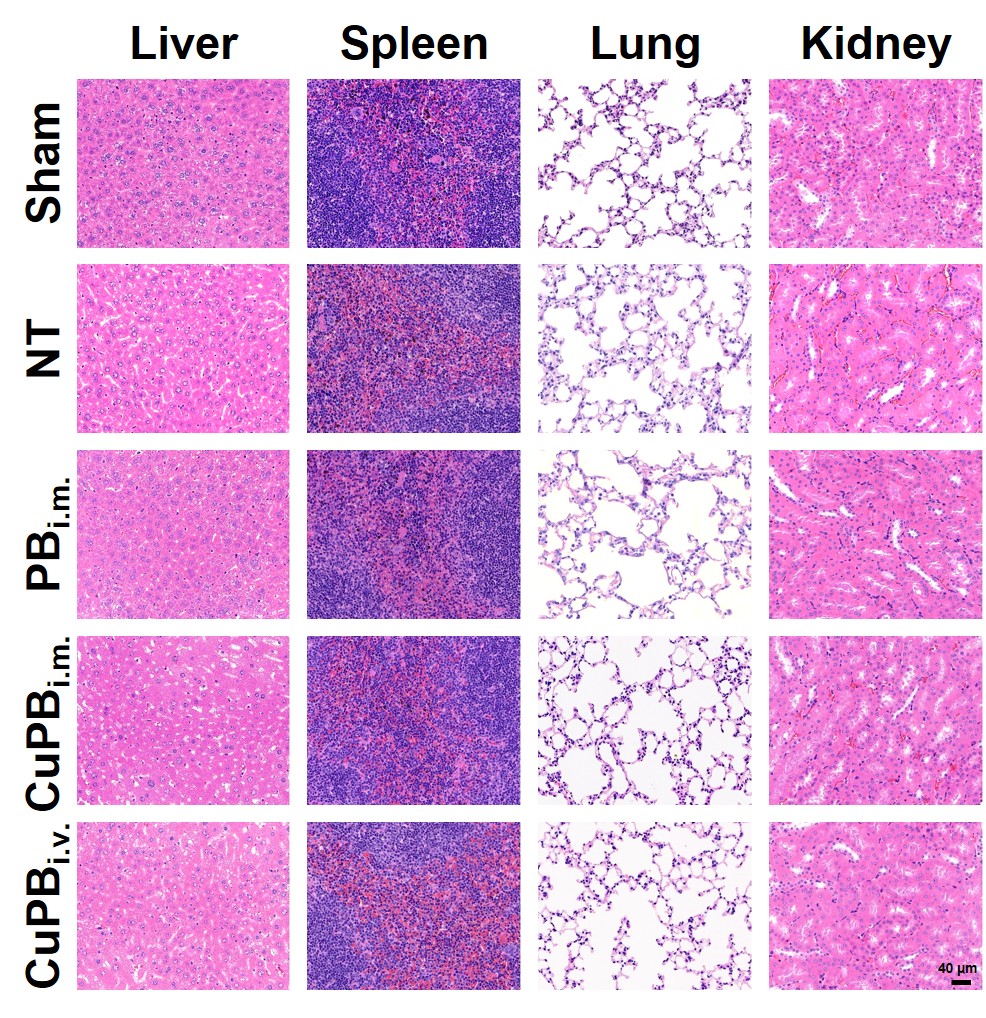


**Figure S30.** *In vivo* safety assessment of CuPB nanozymes treatment in mice with myocardial infarction.

**Table S1.** Particle size distribution of XPB nanozymes determined by DLS.


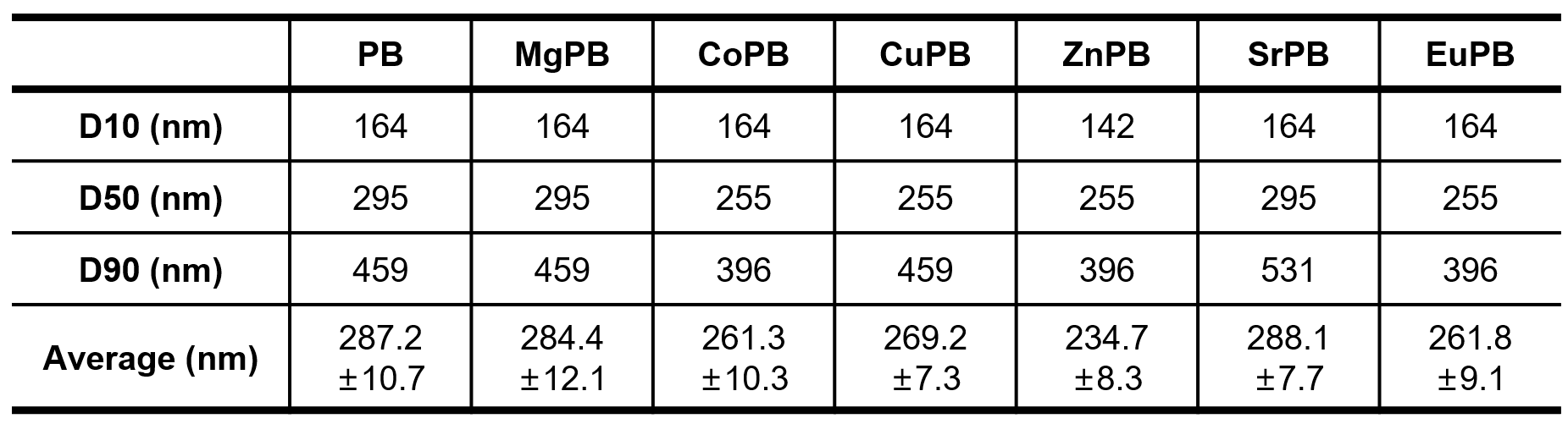

Supplement: Supplementary 1 — Figs. S1 to S30 Table S1 [file research.1260.f1.docx]
